# Sericin promotes chondrogenic proliferation and differentiation via glycolysis and Smad2/3 TGF-β signaling inductions and alleviates inflammation in three-dimensional models

**DOI:** 10.1038/s41598-024-62516-y

**Published:** 2024-05-21

**Authors:** Kamonpan Fongsodsri, Wacharaporn Tiyasatkulkovit, Urai Chaisri, Onrapak Reamtong, Poom Adisakwattana, Suangsuda Supasai, Tapanee Kanjanapruthipong, Passanesh Sukphopetch, Pornanong Aramwit, Sumate Ampawong

**Affiliations:** 1 Department of Tropical Pathology, Faculty of Tropical Medicine, Mahidol University, 420/6 Ratchawithi Road, Ratchathewi, Bangkok, 10400 Thailand; 2 Department of Biology, Faculty of Science, Chulalongkorn University, Bangkok, 10330 Thailand; 3 Department of Molecular Tropical Medicine and Genetics, Faculty of Tropical Medicine, Mahidol University, Ratchawithi Road, Ratchathewi, Bangkok, 10400 Thailand; 4 Department of Helminthology, Faculty of Tropical Medicine, Mahidol University, Ratchawithi Road, Ratchathewi, Bangkok, 10400 Thailand; 5 Department of Microbiology and Immunology, Faculty of Tropical Medicine, Mahidol University, Ratchawithi Road, Ratchathewi, Bangkok, 10400 Thailand; 6 Bioactive Resources for Innovative Clinical Applications Research Unit and Department of Pharmacy Practice, Faculty of Pharmaceutical Sciences, Chulalongkorn University, Phayathai Road, Pathumwan, Bangkok, 10330 Thailand; 7 The Academy of Science, The Royal Society of Thailand, Dusit, Bangkok, 10330 Thailand

**Keywords:** Sericin, Proliferation, Differentiation, Glycolysis, TGF-β signaling, Cell biology, Cell signalling, Mechanisms of disease, Chronic inflammation, Experimental models of disease

## Abstract

Knee osteoarthritis is a chronic joint disease mainly characterized by cartilage degeneration. The treatment is challenging due to the lack of blood vessels and nerve supplies in cartilaginous tissue, causing a prominent limitation of regenerative capacity. Hence, we investigated the cellular promotional and anti-inflammatory effects of sericin, *Bombyx mori*-derived protein, on three-dimensional chondrogenic ATDC5 cell models. The results revealed that a high concentration of sericin promoted chondrogenic proliferation and differentiation and enhanced matrix production through the increment of glycosaminoglycans, COL2A1, COL X, and ALP expressions. *SOX-9* and *COL2A1* gene expressions were notably elevated in sericin treatment. The proteomic analysis demonstrated the upregulation of phosphoglycerate mutase 1 and triosephosphate isomerase, a glycolytic enzyme member, reflecting the proliferative enhancement of sericin. The differentiation capacity of sericin was indicated by the increased expressions of procollagen12a1, collagen10a1, rab1A, periostin, galectin-1, and collagen6a3 proteins. Sericin influenced the differentiation capacity via the TGF-β signaling pathway by upregulating *Smad2* and *Smad3* while downregulating *Smad1*, *BMP2*, and *BMP4*. Importantly, sericin exhibited an anti-inflammatory effect by reducing IL-1β, TNF-α, and MMP-1 expressions and accelerating COL2A1 production in the early inflammatory stage. In conclusion, sericin demonstrates potential in promoting chondrogenic proliferation and differentiation, enhancing cartilaginous matrix synthesis through glycolysis and TGF-β signaling pathways, and exhibiting anti-inflammatory properties.

## Introduction

Osteoarthritis (OA) is a degenerative joint disease, which is the most common form of arthritis^1^. It causes chronic pain, disability, and reduction of joint motion and function^2^. OA is commonly found in the knees, hands, and hips, and it affects over 250 million people worldwide^1,3^. In particular, knee osteoarthritis contributes the highest burden^4^. The global prevalence of knee osteoarthritis has increased in recent decades and continues to rise due to the aging population, obesity, and other factors^5^. Indeed, the increase in the prevalence of this disease may cause a significant burden on the worldwide healthcare system^5^. Articular cartilage is a hyaline cartilage consisting mainly of chondrocytes and a dense extracellular matrix (ECM)^6^. ECM predominantly comprises type II collagen, proteoglycans, and other minor components^6^. In addition, chondrocytes play a crucial role in maintaining cartilage homeostasis through proliferation and secretion of their matrix and matrix-degrading enzymes^7^. During OA, these normal processes are altered, significantly affecting cartilage homeostasis. Chondrocytes change their behavior, resulting in decreased synthesis and increased catabolism of ECM, which accelerates ECM degradation in cartilage^8,9^. Subsequently, chondrocytes secrete inflammatory cytokines such as IL-1β, TNF-α, and IL-6, as well as matrix metalloproteinases (MMPs) including MMP-1, MMP-3, and MMP-13, and aggrecanases^7,10^. The primary hallmark of OA is the degradation of ECM, particularly type II collagen and proteoglycan, with glycosaminoglycan degradations accompanied by a reduction in the number of chondrocytes^7,11^. This occurs as a result of imbalanced chondrocyte activity, characterized by reduced ECM synthesis and increased ECM breakdown. Sericin is a natural product derived from *Bombyx mori* silkworm cocoons^12^. It is a glue-like protein that binds two fibroin filaments, forming the structure of silk yarn^13^. Furthermore, sericin is predominantly composed of polar amino acids such as serine, threonine, and aspartic acid, which confer its distinctive adhesive and gel-forming properties^14^. It can induce collagen production both in vitro and in vivo^15,16^ and has anti-inflammatory effects that suppressed the production of pro-inflammatory cytokines^17^. Interestingly, sericin has been used for cartilage tissue engineering, positively affecting chondrogenic proliferation and viability, including promoting the cartilage-specific ECM components and the expression of specific cartilage genes^18,19^. Several studies have explored the characteristics of sericin in cartilage tissue engineering and repair focusing on healing properties, material designs, chemical, physical and biological properties, and biological safety. However, there needs to be more focus on its specific underlying potential mechanisms that sericin promotes chondrogenesis and knee osteoarthritis treatment.

This study aims to investigate the properties of sericin on chondrogenic proliferation, differentiation, and its specific mechanisms using pellet culture. Additionally, the anti-inflammatory capacity of sericin in the early phase of chondrocyte inflammation was assessed using scaffold culture. Various approaches were performed to explore the effects of sericin, including cytochemistry (alcian blue staining), immunohistochemistry, proteomic analysis, electron microscopy, and RT-qPCR. This study provides a better understanding of the beneficial effects of sericin on the promotional effect on proliferation and differentiation in chondrogenic cells. These findings contribute to the potential development of sericin for therapeutic knee osteoarthritis applications in the future.

## Result

### Cytotoxicity test of sericin on ATDC5 cells

**Figure 1:**
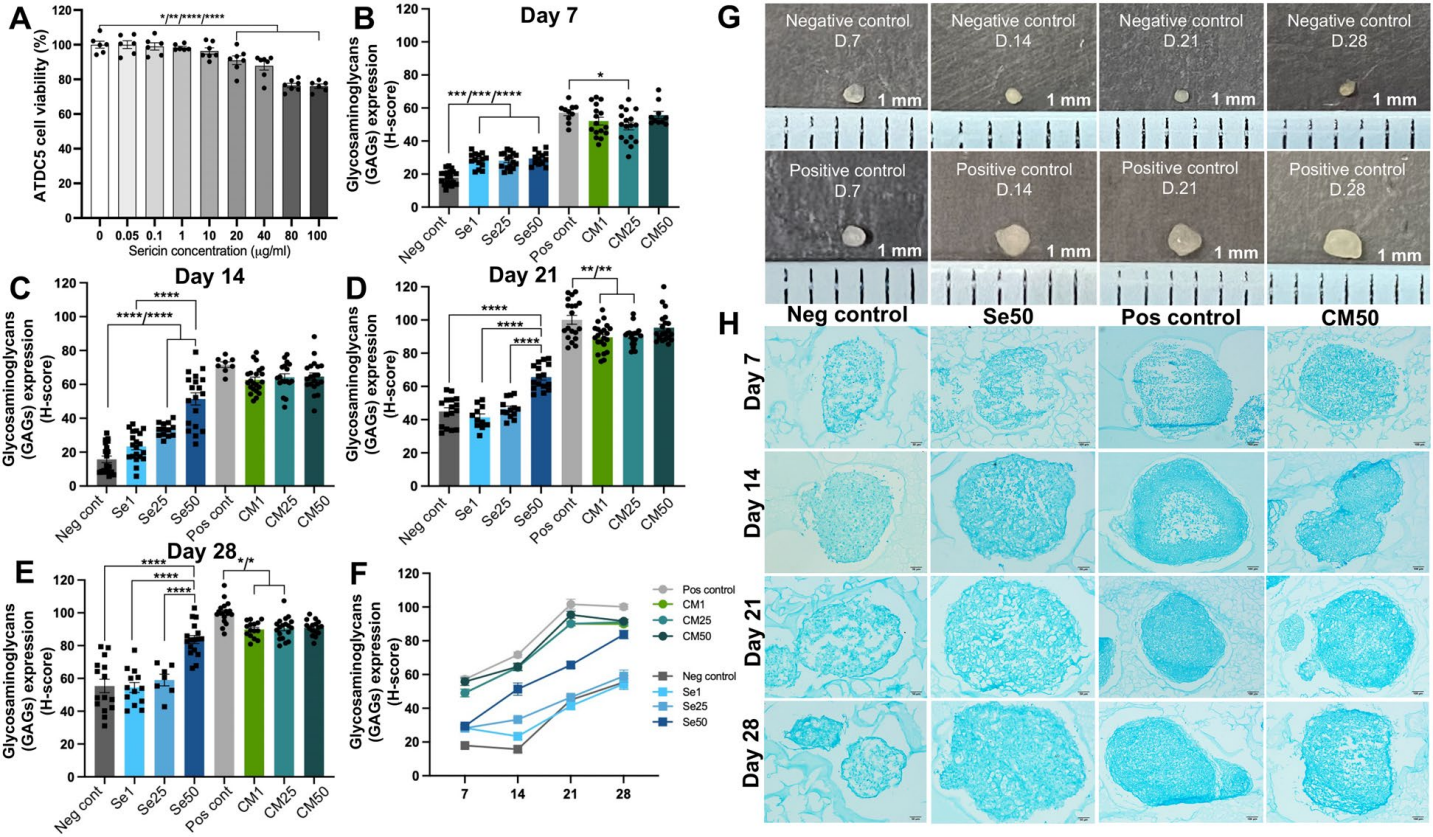
The ATDC5 cell viability and GAGs production (H-score) expression after incubation with sericin in several concentrations. (**A**) Assessment of ATDC5 cell viability treated with various concentrations of sericin for 48 h. The GAGs expression of the negative control, Se1, Se25, Se50, positive control, CM1, CM25, and CM50 (**B**) on day 7, (**C**) day 14, (**D**) day 21, and (**E**) day 28. (**F**) The line graph of GAGs expression in all periods. (**G**) The macroscopic image of control groups on days 7, 14, 21, and 28. (**H**) The alcian blue staining microscopic images of negative control, Se50, positive control, and CM50 groups on day 7, 14, 21, and 28. The abbreviations used in this study were as follows: Se1, Se25, and Se50, which referred to culture medium with 1, 25, and 50 μg/ml of sericin solution, respectively. The CM1, CM25, and CM50 referred to chondrogenic differentiation medium containing 1, 25, and 50 μg/ml of sericin solution, respectively.

The sericin in various concentrations 0, 0.05, 0.1, 1, 10, 20, 40, 80, and 100 μg/ml were provided the percentage of cell viability 100, 100, 99.03, 98.42, 96.37, 91.02, 87.87, 76.46%, and 76.06%, respectively as shown in Fig. 1A. The result showed no significant difference from 0.05 to 10 μg/ml. It significantly decreased in cell viability at 20, 40, 80, and 100 μg/ml of sericin compared with the 0 μg/ml as a negative control (Fig. 1A). However, at 80 and 100 μg/ml, a percentage of cell viability was presented lower than 80%. Therefore, the selection of sericin concentrations to utilize in this study was based on cell viability greater than 80%: 1 μg/ml as a low, 25 μg/ml as a medium, and 50 μg/ml as a high concentration of sericin.

### Effects of sericin on chondrogenic proliferation and differentiation assessed by glycosaminoglycans (GAGs) production

GAGs expression was evaluated to screen sericin’s effect on chondrogenic proliferation and differentiation. On day 7, the expression of GAGs in the Se1, Se25, and Se50 groups was significantly higher than the negative control (Fig. 1B). The CM25 significantly expressed GAGs lower than positive control (Fig. 1B). On day 14, Se25 and Se50 groups significantly increased compared to the negative control (Fig. 1C). Moreover, there was no significant difference between CM1, CM25, CM50, and positive control. On day 21, Se50 group significantly expressed greater than the negative control, Se1, and Se25 groups (Fig. 1D) and no significant difference between the CM50 and positive control. Nonetheless, the CM1 and CM25 groups were significantly lower than positive control (Fig. 1D). On day 28, the GAGs expression in the Se50 group was considerably higher than the negative control, Se1, and Se25 groups (Fig. 1E). The CM50 group had no significant difference from the positive control. In contrast, the CM1 and CM25 groups expressed significantly lower than the positive control group (Fig. 1E).

To summarize these findings, GAG expression in the Se50 group was significantly higher than in the control at every time point. In contrast, GAG expression in the CM50 group did not show a significant difference from the positive control at any time point. All groups were presented the GAGs expression in a line graph from day 7 to day 28 (Fig. 1F). The macroscopic ATDC5 pellets in negative and positive controls on days 7, 14, 21, and 28 were illustrated in Fig. 1G. According to the alcian blue staining result, the Se50, CM50, negative control, and positive control groups on days 7, 14, 21, and 28 were selected to examine cartilage-specific markers in immunohistochemistry. The alcian blue microscopic images of selected groups are presented in Fig. 1H.

### Effect of sericin on enhancement of chondrogenic differentiation assessed by immunohistochemical examination of cartilage-specific markers

**Figure 2:**
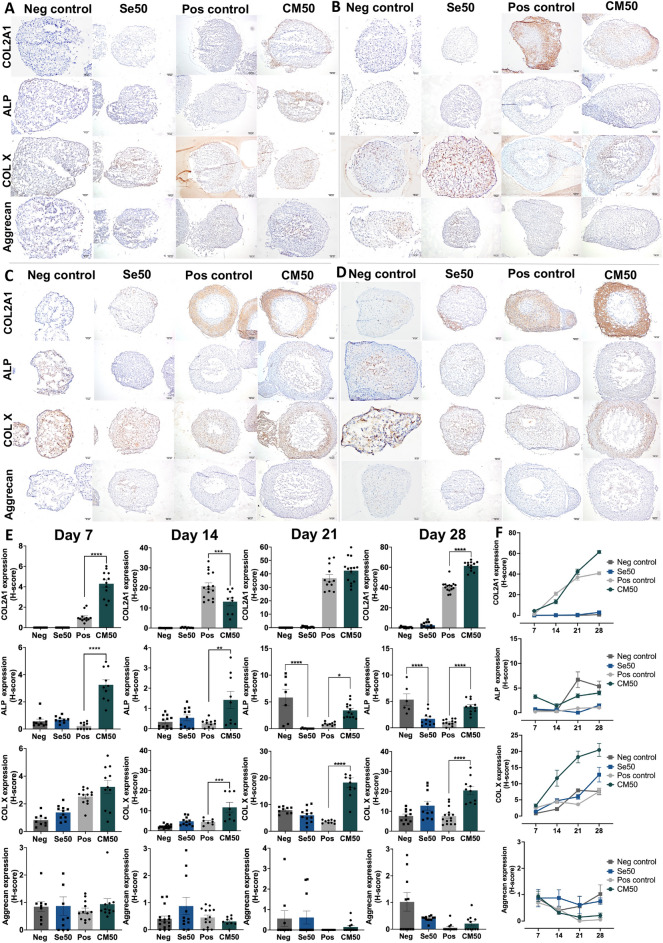
The immunolocalized microscopic images and H-score expression graphs of cartilage-specific markers (COL2A1, ALP, COL X, and aggrecan). The immunolocalized images of the ATDC5 pellet in negative control, Se50, positive control, and CM50 (**A**) on day 7, (**B**) day 14, (**C**) day 21, and (**D**) day 28. (**E**) Bar graphs showing the H-score expression of four specific cartilage markers (COL2A1, ALP, COL X, and aggrecan) on day 7, 14, 21, and 28. (**F**) The line graph of the COL2A1, ALP, COL X, and aggrecan markers from day 7 to 28 across all pellet groups.

Immunohistochemical staining of COL2A1, COL X, and aggrecan indicated the cartilage-specific extracellular matrix components. ALP and COL X are markers of hypertrophic chondrocytes referred to as the chondrogenic differentiation. The results demonstrated that on day 7, COL2A1 expression was absent in the negative and Se50 groups (Fig. 2A, E). The CM50 group had more significantly expressed the COL2A1 and ALP than presented in the positive control (Fig. 2A, E). Moreover, COL X and aggrecan expressions did not differ significantly among the groups (Fig. 2A, E). On day 14, the COL2A1, ALP, COL X, and aggrecan markers were not significantly different in the Se50 group compared to the negative control (Fig. 2B, E). The CM50 group showed significantly lower expression of the COL2A1 marker compared with the positive control. Additionally, the ALP and COL X markers were significantly expressed higher in the CM50 than the positive control (Fig. 2B, E). On day 21, the expression of COL2A1 in both the Se50 and CM50 groups was not significantly different from the controls (Fig. 2C, E). The Se50 groups significantly decreased the ALP compared to the negative control (Fig. 2C, E). In comparison, the expression levels of ALP and COL X were significantly higher in the CM50 group than in the positive control (Fig. 2C, E). However, there were no differences in the aggrecan expression among the pellet groups on day 21 (Fig. 2C, E). On day 28, COL2A1, COL X, and aggrecan expression in the Se50 group were not significantly different from the negative control (Fig. 2D, E). Nonetheless, ALP in Se50 significantly declined compared to the control. In the CM50 group, the expressions of COL2A1, ALP, and COL X were significantly higher than those in the positive control on day 28 (Fig. 2D, E). There was no significant difference in aggrecan marker between CM50 and positive control (Fig. 2D, E).

The summary of this finding throughout the experiment revealed that the CM50 group demonstrated higher expressions of COL2A1, ALP, and COL X than the positive control. In contrast, the Se50 group generally did not exhibit significant differences from the negative control, except for a decrease in ALP expression. Expression of aggrecan remained consistent and did not show significant alteration in comparison with control groups.

According to this result, a high concentration of sericin in the CM50 group significantly promoted chondrogenic differentiation and enhanced the phenotypic chondrocyte prehypertrophy via expression of COL2A1, ALP, and COL X when compared with positive control on day 28. The overview of COL2A1, ALP, COL X, and aggrecan expression levels across all groups from day 7 to day 28 was depicted in the line graph (Fig. 2F). Based on immunohistochemistry results, statistical analysis of the Pearson correlation for four markers from day 7 to day 28 showed that COL2A1 was significantly positively correlated with COL X (0.592, *p* = 0.000) and aggrecan (0.231, *p* = 0.019). In addition, ALP and COL X demonstrated a significant positive correlation (0.377, *p* = 0.000) (Supplementary Table S1).

### Investigation of ultrastructural cytoskeleton proteins in ATDC5 pellets using immunogold labeling

**Figure 3:**
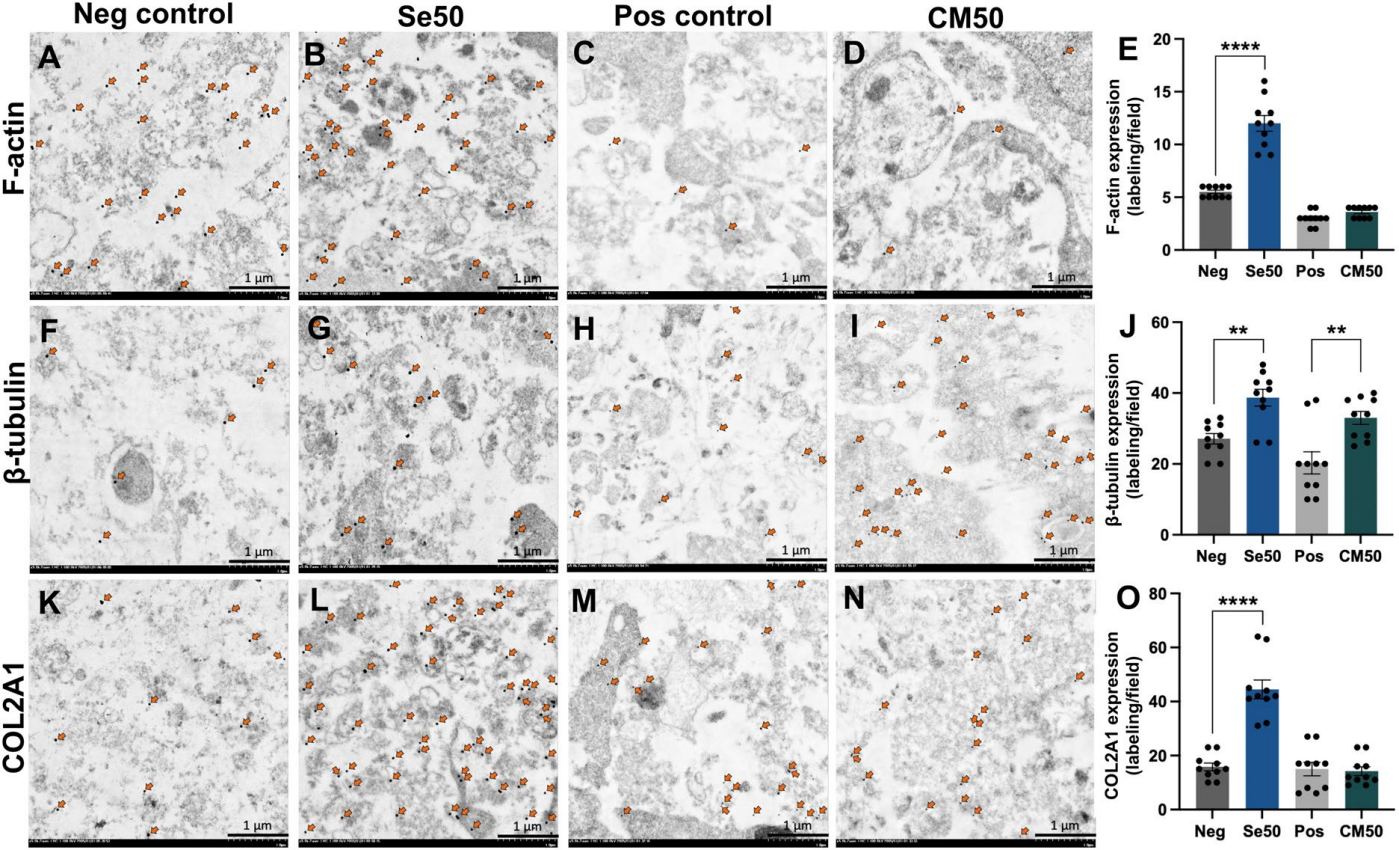
The immunogold labeling of ultrastructural cytoskeleton proteins (F-actin and β-tubulin) and COL2A1 in ATDC5 pellets. The transmission electron microscopic images of pellets from the negative control, Se50, positive control, and CM50 on day 28 were stained with (**A**–**D**) F-actin, (**F**–**I**) β-tubulin, and (**K**–**N**) COL2A1. The bar graphs illustrated the expression of each marker as a labeling/field: (**E**) F-actin, (**J**) β-tubulin, and (**O**) COL2A1.

The ATDC5 pellets on day 28 were examined with the ultrastructural cytoskeleton proteins (F-actin and β-tubulin) and COL2A1 using the immunogold labeling technique (Fig. 3A–O). The result revealed that the expression of F-actin, β-tubulin, and COL2A1 were significantly increased in Se50 compared with the negative control (Fig. 3E, J, and O). Furthermore, CM50 showed significantly higher β-tubulin expression compared to the control groups. (Fig. 3I and J). However, there was no significant difference in F-actin and COL2A1 expressions between CM50 and positive control (Fig. 3E and O).

### Cartilaginous proliferative and differentiative mRNA levels in ATDC5 pellets using RT-qPCR

**Figure 4:**
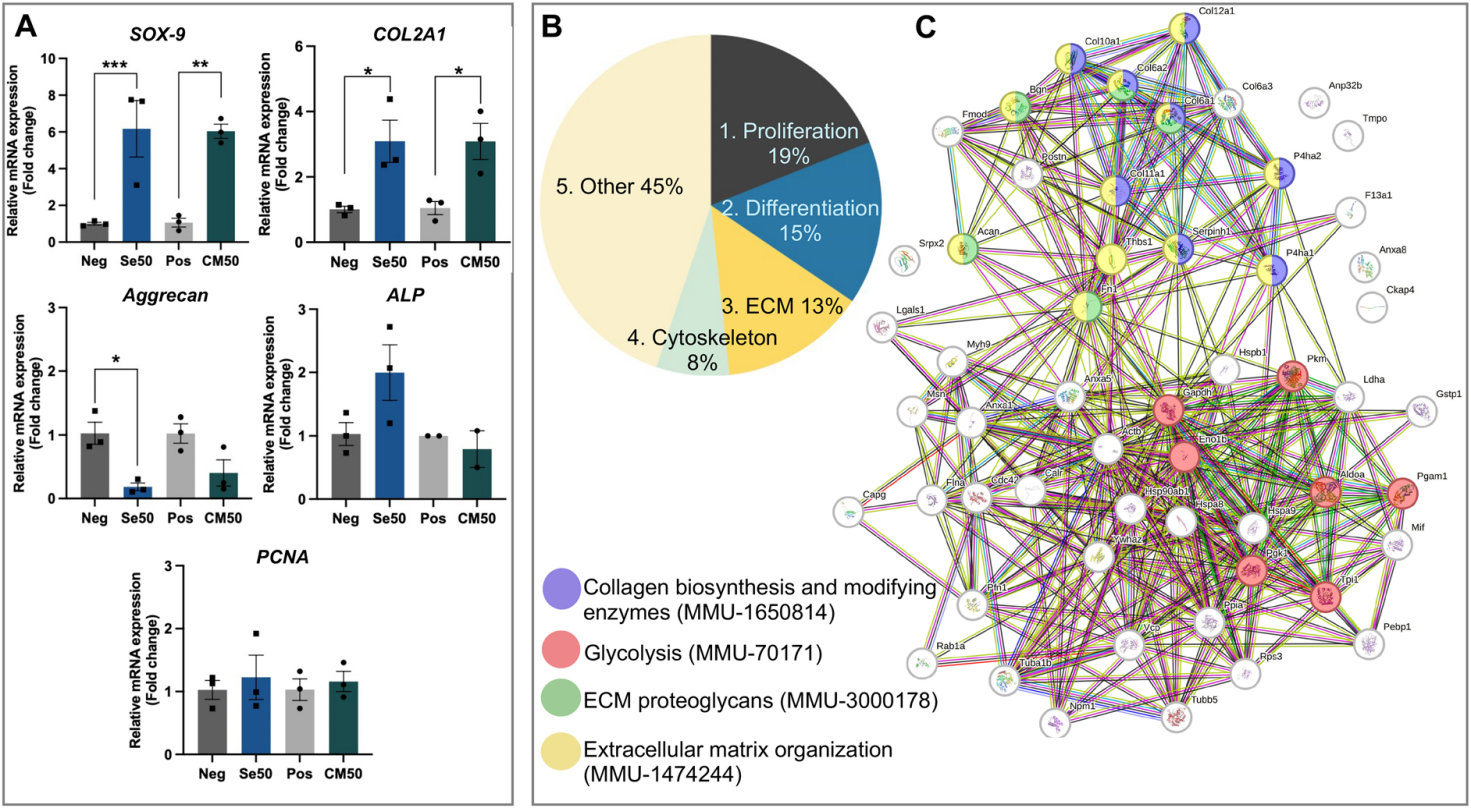
The cartilage specific-gene expression (*SOX-9*, *COL2A1*, *ALP*, *aggrecan*, and *PCNA*) and a proteomic analysis in pellets on day 28. (**A**) The relative gene expression of *SOX-9*, *COL2A1*, *ALP*, *aggrecan*, and *PCNA* in pellets from the negative control, Se50, positive control, and CM50 on day 28. (B–C) The proteomic results were illustrated as follows: (**B**) A pie chart classifying 106 upregulated proteins into five groups associated with chondrogenesis: 1. proliferation of chondrocytes (19%), 2. differentiation of chondrocytes (15%), 3. extracellular matrix proteins (13%), 4. cytoskeletal proteins (8%), and 5. others (45%) and (**C**) A diagram of protein–protein interactions within the 4 categorized groups. The interactions of these proteins are associated with various biological processes: collagen biosynthesis and modifying enzymes (purple; MMU-1650814), glycolysis (red; MMU-70171), ECM proteoglycans (green; MMU-3000178), and extracellular matrix organization (yellow; MMU-1474244).

ATDC5 pellets in the negative control, Se50, positive control, and CM50 groups on day 28 were analyzed for the gene expression related to cartilage-specific chondrocyte proliferation and differentiation using RT-qPCR. The results showed that the gene expression of *SOX-9 and COL2A1* were significantly upregulated in both Se50 and CM50 compared to control groups (Fig. 4A). The *ALP* and *PCNA* expressions were not significantly different in Se50 and CM50 compared to their controls. Nonetheless, the *aggrecan* had downregulated considerably in the Se50 group, and there was no significant difference in the CM50 group compared with the control (Fig. 4A).

### Label-free proteomic analysis of ATDC5 pellets

#### Protein identification and classification

ATDC5 pellets in the CM50 group and positive control on day 28 were performed in a proteomic study to determine the sericin effect on enhancing chondrogenic proliferation and differentiation. The proteomic result showed that the total protein was 487, and significantly upregulated proteins with fold change ≥ 2 were 106 in CM50. Moreover, downregulation was found in 41 proteins with no significant difference (Supplementary Table S2). All 106 significantly upregulated proteins were categorized based on their biological functions, which are closely related to chondrogenesis, chondrogenic proliferation, and differentiation processes. These proteins were grouped into five categories: 1) proliferation (22 proteins, 19%), 2) differentiation (18 proteins, 15%), 3) extracellular matrix (16 proteins, 13%), 4) cytoskeleton (nine proteins, 8%), and 5) other functions (53 proteins, 45%), as shown in Fig. 4B (Supplementary Table S3). It is important to highlight that some proteins may exhibit multiple functions and therefore belong to more than one group. Furthermore, the proteins in 4 major groups (proliferation, differentiation, extracellular matrix, and cytoskeleton groups) from Fig. 4B were explored the protein–protein interaction and functional pathways using the STRING and Reactome databases, respectively. The result of protein–protein interaction and functional pathways were illustrated as a diagram in Fig. 4C. These protein interactions were associated with collagen biosynthesis and modifying enzymes (purple; MMU-1650814), glycolysis (red; MMU-70171), ECM proteoglycans (green; MMU-3000178), and extracellular matrix organization (yellow; MMU-1474244) (Fig. 4C).

#### The differentiation proteins and their functions

The top 10 upregulated proteins with a fold change of ≥ 10 were detailed in Table 1, along with their biological functions. Phosphoglycerate mutase 1 had the highest fold change, followed by polyubiquitin C, triosephosphate isomerase, procollagen type XII alpha 1, collagen-alpha-1 type X, rab1A, periostin, profilin-1, galectin-1, and collagen-alpha-3(VI) chain. Additionally, some proteins play multiple roles. For example, collagen-alpha-1 type X is an ECM protein that indicates differentiation in chondrocytes. Rab1A is involved in chondrogenic proliferation and differentiation. Another ECM protein, Periostin is specific to hypertrophic chondrocytes. Profilin-1 is an actin monomer-binding protein that plays an important role during chondrocyte division.

**Table 1:** The list of upregulated ten proteins associated with chondrogenic proliferation and differentiation.

Protein alteration	*p* value	Fold change	Protein accession	Protein name	Protein function	Protein groups	Sequence coverage (%)
Up-regulation	0.000050	18.10	PGAM1_MOUSE	Phosphoglycerate mutase 1	Phosphoglycerate mutase 1 is a crucial enzyme in glycolysis which response to energy production and nucleotide biosynthesis^20^. It changes 3-phosphoglycerate (3-PG) to 2- phosphoglycerate (2-PG)^20^	1	40.6
	0.001352	16.85	UBC_MOUSE	Polyubiquitin C	Polyubiquitin C is a ubiquitin protein and involved in protein modification (ubiquitination) ^#^	5	42.2
	0.000019	14.46	TPIS_MOUSE	Triosephosphate isomerase	Triosephosphate isomerase is an efficient glycolytic enzyme in cartilage metabolism by catalyzing glycerone phosphate or dihydroxyacetone phosphate (DHAP) and D-glyceraldehyde-3-phosphate (GA3P)^21^	1	48.8
	0.010625	12.99	COCA1_MOUSE	Procollagen, type XII, alpha 1	Procollagen12a1 is involved in col12a1 formation, which is a minor collagen in the extracellular matrix of articular cartilage^22^	3	23.8
	0.000083	12.17	COAA1_MOUSE	Collagen-alpha-1 type X	Collagen10a1 or collagen X is the extracellular matrix which exclusively synthesized by hypertrophic chondrocytes and found in the calcified zone of articular cartilage^22^	2,3	18.5
	0.000838	11.68	RAB1A_MOUSE	Rab1A	Rab1A is a small GTPase and recruits mTORC1 under amino acid simulation at the Golgi^23^. mTORC1 regulates chondrocyte proliferation and differentiation^24^	1,2	10.7
	0.000002	11.50	POSTN_MOUSE	Periostin	Periostin is an extracellular matrix protein and coordinates cell adhesion and differentiation^25^. It has been identified as a gene specific to dark hypertrophic chondrocytes^26^	2,3	31.4
	0.000242	11.08	PROF1_MOUSE	Profilin-1	Profillin-1 is an actin monomer-binding protein which plays a crucial role in dynamic rearrangements of the actin cytoskeleton during chondrocyte division^27^	1,4	43.1
	0.000037	10.77	LEG1_MOUSE	Galectin-1	Galectin-1 mediates the chondrocytes interact to semi-synthetic glycopolymer which stimulates the chondrocyte aggregation production of collagen type2 and GAGs^28^	2	17.8
	0.007617	10.73	Q9Z0I9_MOUSE	Collagen alpha-3(VI) chain	Col6a3 a specialized pericellular matrix (thin layer surrounds chondrocytes)^22^	3	28.2

The proteins were classified into five groups: 1. Proliferation of chondrocyte, 2. Differentiation of chondrocyte, 3. Extracellular matrix, 4. Cytoskeletal proteins, and 5. Others. ^#^in the table represented a basic function from the UniProt database (www.uniprot.org).

#### Sericin induced chondrogenic proliferation via the upregulation of glycolytic proteins

**Figure 5:**
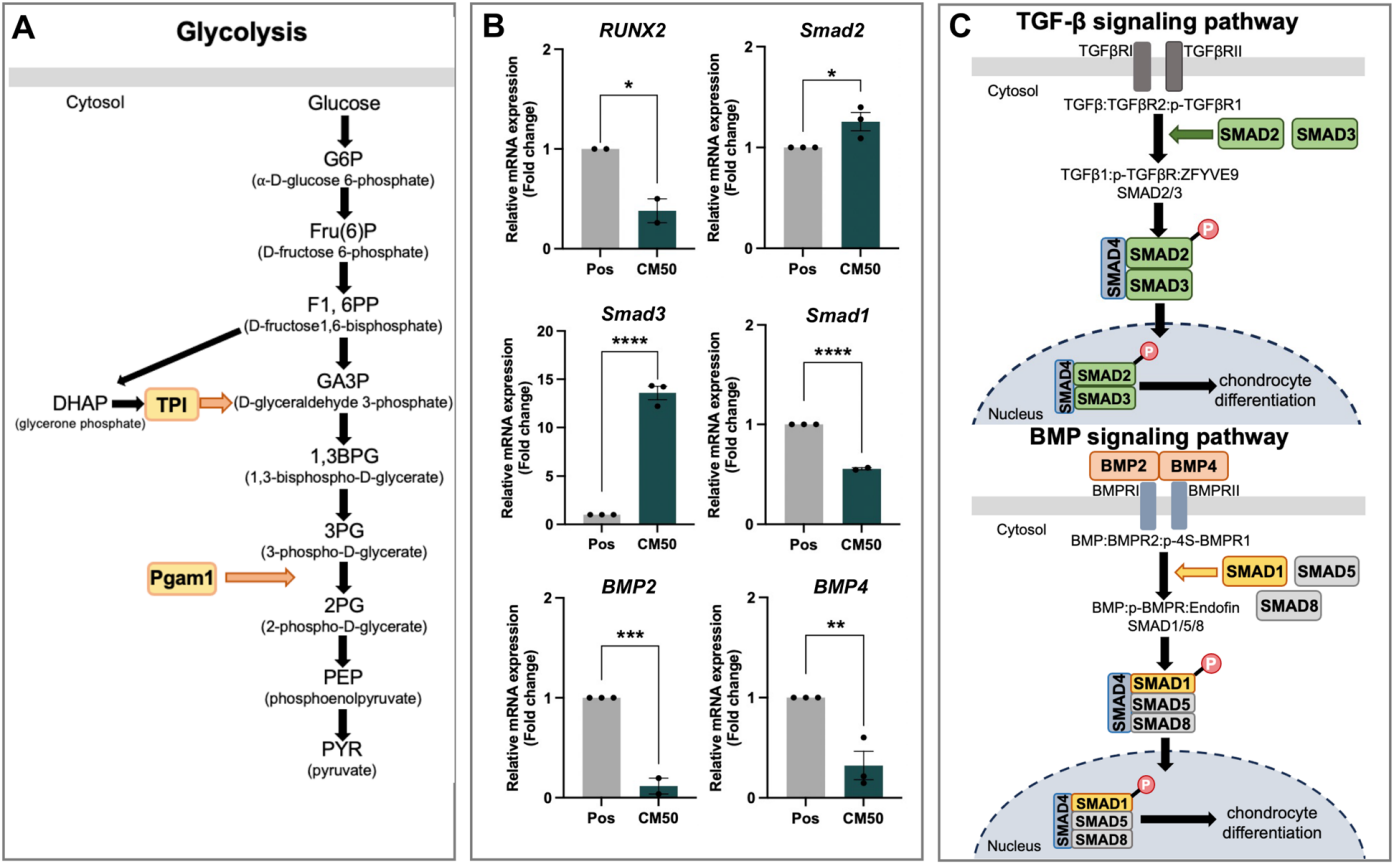
The schematic of glycolysis, exploration of chondrogenic differentiation via gene expression of TGF-β and BMP signaling, and illustration of TGF-β and BMP signaling pathways. (**A**) The presence of glycolytic enzymes (PGAM1 and TPI) in the glycolysis pathway, slightly adapted from the Reactome database (MMU-70171). (**B**) Relative gene expression of *Smad2* and *Smad3* (TGF-β signaling), and *RUNX2, Smad1*, *BMP2* and *BMP4* (BMP signaling). (**C**) Illustrations of *Smad2* and *Smad3* (green boxes) in TGF-β signaling pathway (MMU-2173789.1) and the *Smad1*, *BMP2* and *BMP4* (yellow and orange boxes) in BMP signaling pathway (MMU-201451.1), adapted from the Reactome database.

According to the proteomic result found that phosphoglycerate mutase 1 (Pgam1) and triosephosphate isomerase (TPI) were significantly upregulated proteins in CM50, which had fold changes 18.10 and 14.46, respectively (Table 1). The function of Pgam1 converts 3-phosphoglycerate (3-PG) to 2-phosphoglycerate (2-PG), and TPI catalyzes the interconversion of glycerone phosphate (DHAP) and D-glyceraldehyde-3-phosphate (GA3P), as illustrated in Fig. 5A.

### Sericin induced chondrogenic differentiation through TGF-β/Smad signaling pathway using RT-qPCR

A part of the proteomic result showed that procollagen12a1, collagen10a1, rab1A, periostin, galectin-1, and collagen6a3 were upregulated in the CM50 group (Table 1). The expression of these proteins indicated the chondrogenic differentiation was notably promoted. Moreover, the exploration of the specific mechanism involved in chondrocyte differentiation was examined by RT-qPCR. The relative mRNA expression of *Smad2* and *Smad3* in the CM50 group were significantly higher than in the control group (Fig. 5B). In contrast, *RUNX2, Smad1, BMP2*, and *BMP4* expressions had significantly down-expressed in CM50 compared with control (Fig. 5B). This result revealed that sericin significantly promoted the expression of *Smad2* and *Smad3* via TGF-β signaling while downregulating *RUNX2, Smad1, BMP2*, and *BMP4* in BMP signaling. The up-expression of *Smad2* and *Smad3* in TGF-β signaling and the down-expression of *Smad1, BMP2*, and *BMP4* in BMP signaling were illustrated in the schematic mechanisms in Fig. 5C.

### The ultrastructure of gelatin scaffold with or without ATDC5 cell co-culture using SEM

**Figure 6:**
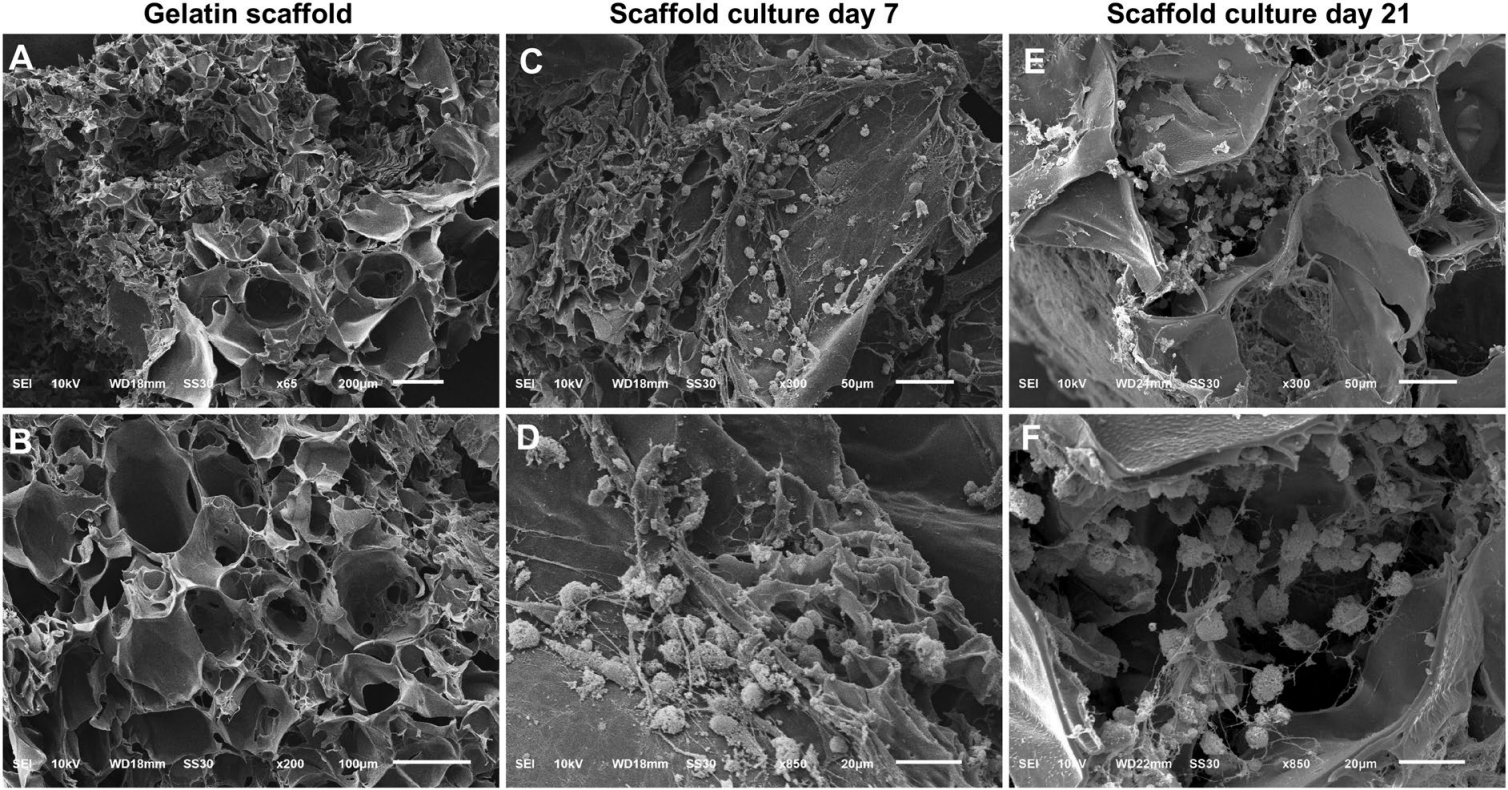
The scanning electron microscopic images of the gelatin scaffold structure and culture. (**A** and **B**) The structure of porous gelatin scaffolds without cells, (**C** and **D**) The scaffold culture with ATDC5 chondrogenic cells on day 7, and (**E** and **F**) day 21.

The ultrastructure of gelatin scaffolds and the attachment of ATDC5 cells throughout days 7 to 21 were examined using a SEM. The gelatin scaffold presented a sponge-like, porous structure with various pore sizes (Fig. 6A and B). The porous scaffold facilitated the infiltration, proliferation, and nutrient transportation of chondrocytes. By day 7 of scaffold culture, the scaffolds allowed cells to infiltrate and distribute within the pores. The cells were able to attach and grow inside the scaffold's pores (Fig. 6C and D). On day 21, the cells continued to exhibit attachment and colonization on the scaffold (Fig. 6E and F).

### Evaluation of sericin treatment in early chondrocyte inflammation using immunohistochemistry and RT-qPCR

**Figure 7:**
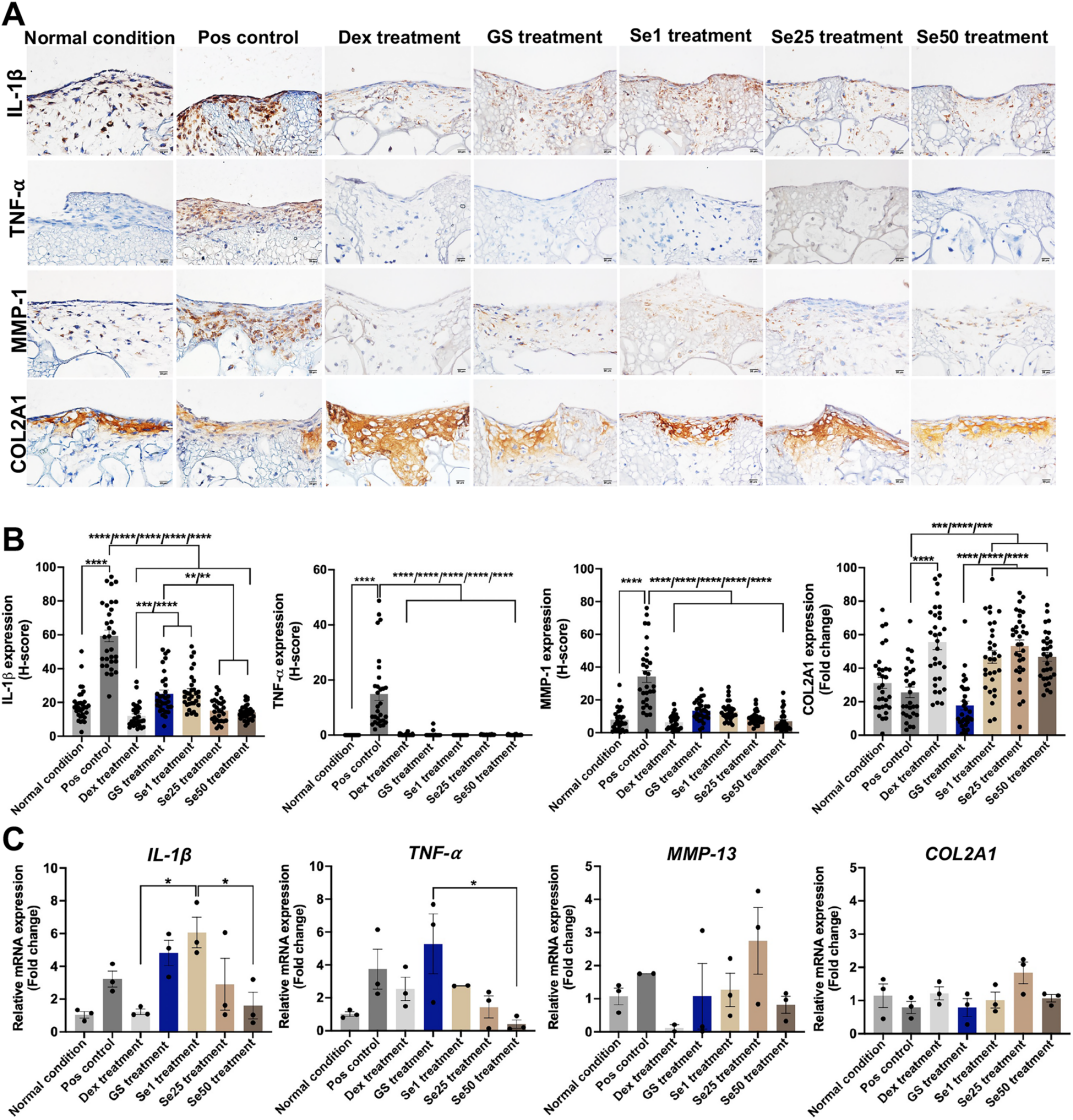
Immunolocalized microscopic images with H-score expression graphs and gene expression analysis of early inflammation markers in scaffold cultures under normal condition, including positive control (inflammation without treatment), Dex treatment, GS treatment, and Se1, Se25, and Se50 treatments. (**A**) The immunolocalized microscopic images of different treatments with early inflammation-related markers (IL1-β, TNF-α, MMP-1, and COL2A1). (**B**) Bar graphs of the H-score expression of IL1-β, TNF-α, MMP-1, and COL2A1 for each treatment group. (**C**) Relative gene expression of *IL-1β*, *TNF-α*, *MMP-13*, and *COL2A*1 in each treatment group. The abbreviations used in this study were as follows: Pos control was the positive control, Dex treatment referred to dexamethasone treatment, GS treatment indicated glucosamine sulfate treatment, and Se1, Se25, and Se50 referred to sericin treatment at 1, 25, and 50 μg/ml concentrations, respectively.

The treatment groups in early inflammation of chondrocytes were investigated for inflammation-related proteins using immunohistochemical staining (Fig. 7A and B). The immunolocalized expressions of IL-1β, TNF-α, and MMP-1 in the positive control group, representing early inflammation, were significantly higher than in the normal condition (Fig. 7A and B). IL-1β exhibited a significant decrease in all treatment groups compared with the positive control. Among the treatment groups, Se25 and Se50 showed significantly lower IL-1β expression than the glucosamine sulfate (GS) treatment (Fig. 7A and B). All treatment groups, particularly those with sericin, significantly reduced TNF-α and MMP-1 expressions compared to the positive control. Notably, the Se1, Se25, and Se50 treatment groups showed a significant elevation in COL2A1 expression compared to the GS treatment, positive control, and normal condition (Fig. 7A and B). Pearson correlation analysis of the immunohistochemistry results showed that IL-1β had significant positive correlations with TNF-α (0.493, *p* = 0.00) and MMP-1 (0.609, *p* = 0.000), in contrast to a significant negative correlation with COL2A1 (− 0.177, *p* = 0.009). COL2A1 expression showed significant negative correlations with IL1-β (− 0.177, *p* = 0.009), TNF-α (− 0.0173, *p* = 0.016), and MMP-1 (− 0.0263, *p* = 0.000) (Supplementary Table S4).

The results of relative gene expression showed that the Se50 and Dex treatments significantly reduced IL-1β gene expression compared to the Se1 treatment (Fig. 7C). The Se50 treatment demonstrated a significantly lower TNF-α gene expression than the GS treatment (Fig. 7C). However, the gene expression levels of *MMP-13* and *COL2A1* were not significantly different across all groups (Fig. 7C).

## Discussion

We investigated the effect of sericin on chondrogenic proliferation and differentiation regarding the specific involved mechanisms and their anti-inflammatory properties using 3D cell culture models by several approaches. In this study, a high concentration of sericin can promote the proliferation and differentiation of chondrocytes through glycolysis and TGF-β signaling pathways characterized by the increase of GAGs production (Fig. 1B–F), stimulation of differentiation and maturation of chondrocytes (COL2A1, ALP, and COL X) (Fig. 2A–F), promoting the essential cytoskeletal proteins (Fig. 3E, J, and O), upregulation of transcription factors *SOX-9* and *COL2A1* ECM genes (Fig. 4A), and activation glycolytic enzymes and differentiation-related proteins as shown in Table 1. In addition, sericin can diminish inflammation via the downregulation of IL1-β, TNF-α, and MMP-1 (Fig. 7A and B).

GAGs play an important role in cartilage homeostasis and act as signaling mediators in several processes, such as cell proliferation, differentiation, migration, and adhesion^29^. The alteration of GAGs structure and components in proteoglycan is an early event in OA, leading to cartilage degradation^30^. Our study demonstrated that a high sericin concentration promote the GAGs production compared with the controls (Fig. 1B–F). The GAGs such as hyaluronic acid and chondroitin sulfate improved in a collagen–sericin scaffold study for cartilage regeneration^18^. An alternative form of sericin revealed that sericin hydrogel can stimulate GAG production more than the hydrogel control group^19^. In addition, another natural product, such as curcumin, induced the production of GAG in chondrogenic C3H10T1/2 cells^31^. Notably, the Se50 group exhibited significantly higher expression of GAGs compared to the negative control from day 7 to day 28. The Se1, Se25, and Se50 groups were ATDC5 pellets cultured in normal growth medium supplemented with sericin at 1, 25, and 50 µg/ml, respectively. These groups lacked chondrogenic differentiation supplements. This result suggested that a high concentration of sericin may potentially serve as a chondrogenic-induced differentiation factor.

The expression of cartilage-specific markers, such as collagen type 2 (COL2A1), ALP, collagen X (COL X), and aggrecan, was detected in the pellet groups using immunohistochemical staining (Fig. 2A–F). COL2A1 and aggrecan are the ECM referred to as chondrogenic proliferation and differentiation^32^, ALP is alkaline phosphatase for hypertrophic chondrocyte marker^33^, and COL X is a specific collagen expressed by hypertrophic chondrocytes^22^. On day 7, our results showed a significant upregulation of COL2A1 and ALP in the CM50 group. Additionally, the CM50 group revealed a heterogeneous cell population, expressing characteristics of both early and late differentiation markers (Fig. 2A, E, and F). By day 28, the Se50 group exhibited a significant decrease in ALP expression (Fig. 2D, E, and F), suggesting that sericin reduces ALP activity, which may inhibit chondrogenic hypertrophy. Similarly, high concentrations of chondroitin sulfate and hyaluronic acid suppressed ALP activity on days 4, 7, and 14 in ATDC5 cell culture^34^. The COL X was not different between Se50 and control groups. Thus, this property of sericin should be more elucidated in a further study for a better understanding of the specific influence of sericin on ALP activity in chondrogenic cells. The hallmark of OA is the degradation of collagen type II^7,11,35^. Our study revealed that the CM50 group showed a significant increase in COL2A1, ALP, and COL X expressions compared to the positive control on day 28 (Fig. 2D, E, and F). Fabrication of sericin and fibroin reported that sericin hydrogel and fibroin film or scaffolds promote the production of COL2A1^19,36,37^. Moreover, other natural products such as pomegranate fruit extract and avocado/soybean unsaponifiable (ASU) can enhance collagen type 2 synthesis in chondrocytes^38,39^. In contrast to curcumin’s effect on hypertrophic chondrocyte markers, COL X was decreased in the curcumin-supplemented group^31^. Aggrecan expression in our study showed no significant difference between each group and time point. This result suggested that sericin supplementation in the Se50 and CM50 groups may not influence aggrecan expression. Similarly, aggrecan expression was not significantly different from the control during the chondrogenic differentiation of rabbit periosteal cells on day 21^40^. Given these properties, sericin can stimulate the production of specific cartilage ECM, particularly collagen, and accelerate chondrocyte maturation. This positive effect could contribute to alleviating OA in terms of forming new cartilage tissue and endochondral ossification.

F-actin and β-tubulin are key components of the cellular cytoskeleton. Therefore, changes in the expression of these proteins can indicate cytoskeletal reorganization. In our study, the expression of cytoskeletal proteins was detected using the immunogold labeling technique. F-actin was highly expressed in the Se50 group; meanwhile, β-tubulin significantly increased in both the Se50 and CM50 groups, as shown in Fig. 3A–O. The function of actin filament in chondrocytes is to maintain cell shape and its phenotypes^41^. β-tubulin is involved in cell division and growth^42^. Moreover, it has been reported that loss of actin, tubulin, and vimentin organization can be found in OA chondrocytes^41,43^. This property indicated that sericin could enhance cytoskeletal protein expressions, which could maintain chondrogenic phenotypes and cell division, thereby improving the cytoskeletal organization of chondrocytes.

The relative gene expression-related chondrogenic proliferation and differentiation markers such as *SOX-9*, *COL2A1*, *aggrecan*, *ALP*, and *PCNA* were observed using RT-qPCR. Interestingly, our result demonstrated that the gene expression of *SOX-9* and *COL2A1* significantly increased in both sericin groups (Se50 and CM50) than the controls (Fig. 4A). SOX-9 is a transcriptional factor that regulates the proliferation and multistep differentiation of chondrocyte^44^. SOX-9 activates the gene expressions in chondrocytes such as *COL2A1, COL9A1, COL11A2,* and others^45^. A mutation in SOX-9 disrupts the chondrogenic differentiation process, resulting in the failure of SOX-9 target gene expression, affecting human skeletal formation, and leading to malformation syndrome^44^. Similar to our study results, silk fibroin film significantly upregulated the gene expression of *SOX-9* compared to the control^46^. Another marker that had significant upregulation in the sericin group of our study is *COL2A1* gene expression. COL2A1 is a major component in cartilage ECM and maintains physiological homeostasis^47^. Degradation and loss of COL2A1 are the typical pathological alterations and frequent observations in OA cartilage^48^.

ASU treatment significantly increased the mRNA level of *COL2A1*^49^. In contrast to our study, curcumin supplementation had no effect on *SOX-9* and *COL2A1* gene expressions^31^. Nonetheless, aggrecan was down-expressed in Se50 compared to the control group in this study (Fig. 4A). According to this result suggested that sericin may contain factors that elevate the gene expressions of *SOX-9* and *COL2A1*, but have a different effect on the gene expression level of aggrecan. However, the specific cellular signaling pathways or molecular epigenetic mechanisms may affect the gene regulation of aggrecan. These influences are unclear and should be elucidated in further studies. Additionally, we found that sericin did not affect to *PCNA* marker on day 28 (Fig. 4A). PCNA is a marker of cells in the proliferative phase of the cell cycle which is often distributed in the S-phase^50^. The collection period of pellets might affect the expression of *PCNA* due to chondrogenic cells on day 28 were fully differentiated cells, reduced proliferation, and entered a quiescent state. *PCNA* expression should be detected during the early stage of cell proliferation.

The proteomic study analyzed the specific proteins and mechanisms associated with chondrogenic proliferation and differentiation in this study. Our proteomic result demonstrated glycolytic enzymes were the most significantly upregulated proteins (Table 1 and Fig. 5A). Glycolysis is a central metabolic pathway involving glucose metabolism and producing cellular energy. It is a major metabolism in chondrocytes for energy production^51^. In addition, the process of cell proliferation demands a significant amount of energy^52^. Pgam1 enzyme is the highest fold change protein, followed by the TPI enzyme in our finding. These enzymes are important in glycolysis for the conversion of critical glycolytic intermediates. It is crucial in several biological processes, particularly energy production and nucleotide biosynthesis^20,53^. Pgam1 activity upregulates various tumor growths by promoting cell proliferation^54^. Pgam1 knockdown in glioma suppressed cell proliferation and enhanced apoptosis via the S-phase cell cycle^55^. Therefore, the CM50 group showed a significant upregulation of proteins involved in glycolysis, which promoted chondrogenic proliferation This finding was consistent with the high expression of COL2A1 observed in immunological staining (Fig. 2D, E, and F), β-tubulin expression in immunogold labeling (Fig. 3I and J), and the gene expressions of *SOX-9* and *COL2A1* (Fig. 4A). Other proteomic studies, Pgam1 and TPI metabolic enzymes are upregulated during chondrogenesis^56^. Proteomic in human OA chondrocytes showed that downregulation of proteins in glycolysis, such as glyceraldehyde 3-phosphate dehydrogenase, enolase, and fructose biphosphate aldolase down-expressed compared to normal chondrocytes^57^.

To investigate the effect of sericin on chondrocyte differentiation, the proteomic study revealed the upregulation of procollagen12a1, collagen10a1, rab1A, periostin, galectin-1, and collagen6a3 proteins. These proteins indicated chondrocyte differentiation. However, the specific mechanism promoted by sericin was examined through relative gene expression analysis. The Smad2/3-dependent TGF-β signaling was significantly increased, indicated by the high levels of *Smad2* and *Smad3* gene expression (Fig. 5B and C). On the contrary, BMP signaling significantly downregulated through decreased gene expressions of *RUNX2*, *Smad1*, *BMP2*, and *BMP4* (Fig. 5B and C). TGF-β signaling controls cartilage homeostasis and development^58^. Several transcriptional factors could regulate chondrocyte proliferation and differentiation. The Smad2/3-dependent TGF-β signaling consists of phosphorylated R-Smads (Smad2/3) and co-Smad4, which transfer into the nucleus to regulate the expression of SOX-9 and RUNX2^59^. By the *SOX-9* gene expression, *SOX-9* significantly increased, while *RUNX2* decreased in the CM50 group compared to the control (Fig. 4A and 5B). Furthermore, the stabilization of SOX-9 protein levels can be modulated by TGF-β. SOX-9 and TGF-β work synergistically to protect chondrocyte function^60^. Nonetheless, RUNX2 expression is suppressed by TGF-β/Smads (Smad2/3), leading to decreased ECM degradation in the chondrocyte^61^. A natural component extracted from *Rhodiola Rosea* called salidroside can promote articular chondrocyte proliferation, *SOX-9, Acan, Col2A1,* and *Col1A1* gene expressions via TGF-β/Smad3 signaling^62^. Smad2 and Smad3 were reported to regulate both chondrocyte proliferation and differentiation in cartilage growth plates^63^. Another member in the TGF-β superfamily is BMP signaling^64^. In our study, BMP signaling was decreased in the sericin group. TGF-β1 induces BMP2 expression to enhance the proliferation of chondrocytes^64^. On the other hand, the inhibition of TGF-β-induced Smad activity may be stimulated by BMP2^64^. Additionally, Smad3 deficiency results in the suppression of TGF-β signaling and activation of BMP2 signaling accelerated chondrocyte maturation and OA^65^. This result suggested that sericin promotes chondrocyte differentiation via upregulation of the Smad2/3-dependent TGF-β signaling pathway, supported by the observed increase of COL2A1 and COL X expression in immunological staining (Fig. 2D, E, and F), β-tubulin expression in immunogold labeling (Fig. 3I and J), and *SOX-9* and *COL2A1* gene expressions (Fig. 4A) as well as the expression of specific cartilage proteins (Table1).

IL-1β and TNF-α cytokines play crucial roles in OA development and progression^66^. During KOA, chondrocytes excessively secrete major cartilage matrix-degrading enzymes, such as MMP-1 (collagenase-1) and MMP-13 (collagenase-3), which efficiently degrade type II collagen^7,10^. In the present study, we emphasize the impact of sericin treatment on chondrocyte inflammation. ATDC5 chondrocytes were cultured within gelatin scaffolds under early inflammation conditions induced by IL-1β and TNF-α. Various treatments were applied, and the cells were investigated using immunological staining and gene expression analysis (Fig. 7A, B, and C). After treatment with sericin illustrated that Se1, Se25, and Se50 treatments significantly reduced the IL-1β, TNF-α, and MMP-1 expressions, including significantly increased the expression of COL2A1. Unfortunately, the Se1, Se25, and Se50 treatments did not show significant differences in the gene expression levels of IL-1β, TNF-α, MMP-13, and COL2A1 compared to the positive control (untreated group) (Fig. 7C). Although studies on the direct effects of sericin treatment specifically on chondrocyte inflammation or OA have been limited. There are many studies of sericin on anti-inflammatory properties in other models^17,67^. Sericin-coated film reduced IL-1β and TNF-α levels in rat psoriasis model^67^. Moreover, sericin downregulated *COX-2*, *iNOS*, *IL-1β*, *IL-6*, *IL-18*, *CCL2*, and *CCL5* gene expressions^17,68^. Other promising effects of natural products for chondrocyte inflammation and OA include curcumin^69^, ASU^49,70^, Danshen^71^, and Icariin^72^. Curcumin inhibited nitric oxide, IL-6, IL-8, and MMP-3 production by IL-1β stimulation^69^. ASU decreased IL-1β, TNF-α, COX-2, and iNOS expression^70^ Danshen diminished the expression of MMP-9 and MMP-13 and promoted Tissue inhibitor of metalloproteinase 1 (TIMP-1) and TIMP-2 expressions^71^. In addition, sericin and curcumin synergize the treatment of inflammation by modulating expressions of IL-4 and IL-10 in *vivo* and in *vitro*^73^. Dexamethasone is a corticosteroid used to treat chondrocyte inflammation and OA studies for decades^74^. Our study, Dex treatment demonstrated that 100 μM of dexamethasone can significantly reduce the expressions of IL-1β, TNF-α, and MMP-1 while promoting the expression of COL2A1 (Fig. 7A and B). In other studies, dexamethasone decreased IL-1β and MMP-1, MMP-3 and MMP-13 expressions^74,75^. Another treatment, glucosamine sulfate (GS) treatment, was set as the standard treatment in this study. GS is an amino-monosaccharide and a natural composition of long-chain glycosaminoglycans in the human cartilage matrix^76^. It is extensively utilized in the treatment of OA^77^. In the present study, GS treatment decreased the expressions of IL-1β, TNF-α, and MMP-1 (Fig. 7A and B). However, it did not promote the expression level of COL2A1, as presented in Fig. 7B. Glucosamine treatment at 100 μM slightly inhibited the IL-1β expression mediated type 2 collagen and MMP-13 in OA chondrocytes and hMSCs^78^. In our study, the gene expression level of GS was not different from the positive control across all markers (Fig. 7C). Treatment with GS alone and GS co-incubated with IL-1β in human OA chondrocytes showed a decrease in the expression of IL-1β, TNF-α, IL-6, MMP-1, MMP-3, and MMP-13, and an increase in COL2A1 expression^76^. Limitations of this study, another method for validating cell proliferation, such as counting the number of cells, should be considered alongside the investigation of various factors for chondrogenic differentiation. Although chondrocyte-destructive factors, MMP-1 and MMP-13, were analyzed in our study, other inhibitory factors such as TIMPs should also be considered to fully understand ECM turnover.

In conclusion, our research team has utilized sericin in several biomedical science approaches in previous studies, such as wound healing, antipsoriasis properties, and anticholesterolemic and hepatoprotective agents. In this study, we conducted an in vitro 3D model demonstrating that sericin reduces inflammation and stimulates collagen production, which can also be beneficial for healing and exerting anti-inflammatory effects. We found that a high concentration of sericin can promote the glycolysis pathway and Smad2/3 TGF-β signaling to stimulate chondrogenic proliferation and differentiation, enhance the cartilaginous matrix synthesis, particularly the collagen productions, and attenuate early inflammation in chondrocytes. These potential mechanisms contributed to elevating GAGs production, COL2A1, COLX, ALP expressions, *SOX-9* and *COL2A1* gene expressions. However, sericin did not affect aggrecan expression which was different from the other positively affected collagen production. Additionally, a high sericin concentration was able to reduce IL-1β, TNF-α, and MMP-1 expressions. Therefore, this finding suggested that sericin exhibits a positive chondrogenic promotional effect and therapeutic potential for osteoarthritis treatment. These findings will be preliminary data for further preclinical research on an in vivo scale in term of its efficacy, application, and degradation rate.

## Materials and Methods

### Sericin extraction

Silk sericin was extracted from *Bombyx mori* cocoons purchased from Chul Thai Silk Co. Ltd., Phetchabun province, Thailand. The method is described in Ampawong and Aramwit’s 2017 study^79^. Cocoon shells of *B. mori* were heated in deionized water by autoclaving at 120 °C for 60 min. The shells were discarded, and the supernatant was filtered to remove fibroin. The sericin extract was analyzed for amino acid composition as a quality control by Central Laboratory (Thailand) Co., Ltd., Bangkok, Thailand.

### Cytotoxicity effect of sericin on ATDC5 chondrogenic cells

The silk sericin solution examined the cytotoxicity effect using MTT (3-(4,5-dimethylthiazol-2-yl)-2,5-diphenyltetrazolium bromide) assay. ATDC5 cells were seeded 5,000 cells/well in 96 well plates and incubated at 37 °C with 5% CO_2_ for 24 h. The sericin was added in varied concentrations at 0, 0.05, 0.1, 1, 10, 20, 40, 80, and 100 μg/ml and incubated for 48 h. After incubation, an MTT solution was added and incubated for 2 h. Dimethylsulfoxide (DMSO) was used to dissolve the insoluble formazan in each well. Then, they measured the absorbance at 570 nm using a microplate reader (synergy H1, Biotek). The selected concentrations of sericin were based on the percentage of cell viability above 80% as non-cytotoxicity^80^.

### Chondrogenic proliferative and differentiative models

#### ATDC5 cell culture

To propagate chondrogenic cells for use in three-dimensional culture, an ATDC5 cell culture was performed. Murine chondrogenic ATDC5 cells (ECACC, England) were cultured as monolayer culture in Dulbecco’s Modified Eagle’s Medium/Nutrient Mixture F-12 (DMEM/F-12) (Gibco, USA), 1% penicillin and streptomycin (10,000 U/mL) (Gibco, USA), and 5% fetal bovine serum (FBS) (Gibco, USA). ATDC5 cells were incubated at 37 °C with 5% CO_2_. The medium was changed twice a week.

#### Three-dimensional pellet culture

A three-dimensional pellet culture was performed to evaluate sericin’s chondrogenic proliferative and differentiative properties in complex cell formation**.**

ATDC5 cells at density 5 × 10^5^ cells/ml in a 15 ml tube were centrifuged 400 × g for 10 min at 4 °C for cell aggregation and formation as a pellet using the pellet culture technique. The pellet cells were incubated in a medium for 3 days at 37 °C with 5% CO_2_. The medium was renewed every 2–3 days. The cap 15 ml tube was slightly unscrewed for gas exchange. The chondrogenic differentiation medium in this study was described from the study of Tare et al.^81^. It comprised DMEM/F-12 (Gibco, USA), 5% FBS, 1% penicillin, and streptomycin (Gibco, USA) supplemented with 1X of ITS premix (Sigma-Aldrich, USA), 10 ng/ml TGF-$\beta$3, 10^−8^ M dexamethasone, and 100 µM ascorbate-2-phosphate.

#### Experimental procedure

The pellets were cultured in (1) the culture medium as a negative control, (2) the culture medium supplemented with sericin solution 1, 25, and 50 μg/ml as a Se1, Se25, and Se50, respectively, (3) the chondrogenic differentiation medium as a positive control, and (4) the chondrogenic differentiation medium supplemented with sericin solution 1, 25, and 50 μg/ml as a CM1, CM25, and CM50, respectively. The pellets were preserved (1) in 4% paraformaldehyde for immunohistostaining, (2) in RNA*later* solution for RT-qPCR, (3) in − 80 °C for proteomic analysis, and (4) in 2.5% glutaraldehyde for electron microscopy.

### 3D-scaffold culture mimicked the early inflammatory model

#### Gelatin scaffold fabrication

Gelatin scaffolds were used in an inflammatory model to explore the anti-inflammatory effect of sericin on chondrogenic cells. A 4% gelatin scaffold was prepared by soaking 4 g of gelatin (Nitta Gelatin Inc, Japan) in 96 ml of distilled water for 15 min to allow for water absorption. Subsequently, they were homogenized at 40 °C for 1 h. The gelatin solution was added to 96 well plates and frozen at − 20 °C overnight. They were lyophilized using the LL3000 freeze-dryer (Thermo Scientific, USA) for 72 h. For the chemical cross-linking, 20 mM of N-(3-Dimethylaminopropyl)-*N*′-ethyl carbodiimide hydrochloride (EDC) (Sigma-Aldrich, USA) and 5 mM of N-hydroxysuccinimide (Acros organics, Belgium) was added into the samples and incubated with shaking for 22 h. The samples were washed four times with shaking by distilled water. They were frozen at − 20 °C for 24 h and lyophilized using a freeze-dryer for 72 h.

#### The observation of the scaffold using a scanning *electron* microscope (SEM)

To characterize the structure of the gelatin scaffold and observe the cell attachment in the scaffold. The gelatin scaffold without cells and with cells on days 7, 14, and 21 were fixed in 2.5% glutaraldehyde in 0.1 M sucrose phosphate buffer (SPB) for 1 h and 1% osmium tetroxide in 0.1 M SPB for 1 h. The pellet samples were dehydrated in a series of ethanol for 10 min in each concentration. The samples were dried using a critical point dryer (CPD) (EM CPD300, Leica®) and coated using a gold sputter coater (Q150R S, Quorum®) for 2 min. The scaffold samples were observed under a SEM (JEOL JSM-6610LV, Japan) with 15 kV acceleration voltages focusing on the ultrastructure of the cell adhesion on the scaffold and scaffold architecture.

#### Experimental protocol

To demonstrate the anti-inflammatory effect of sericin in early inflammation, the scaffold culture mimicked the early inflammation of chondrocytes. In brief, gelatin scaffolds (0.5 × 0.2 cm) were soaked in culture medium overnight. The scaffolds were seeded ATDC5 cells at density 10^6^ cell/15 μl and incubated at 37 °C with 5% CO_2_ for 1 h. They were then transferred into a 24-well plate and the culture medium 1 ml into a well. After three days of incubation, the culture medium was changed to the chondrogenic differentiation medium. The medium was changed every three days, and the cultures were maintained until the end of the experiment on day 21. Early inflammation of chondrocytes was induced on day 14 by adding IL-1β and TNF-α cytokines at 1 ng/ml for 24 h. The inflammatory stimulation was performed twice a week, and treatments were co-incubated with cytokines until day 21. The treatments were applied as follows: dexamethasone at 100 μM, glucosamine at 25 μM, and sericin at 1, 25, and 50 μg/ml. The samples were kept (1) in 2.5% glutaraldehyde for electron microscopy, (2) in 10% NBF for immunohistochemical study, and (3) in RNA *later* solution for RT-qPCR.

### Cytochemistry and immunohistochemistry

The pellet samples were fixed in 4% paraformaldehyde with 15% sucrose overnight. Then, 30% sucrose was used to replace the fixative to preserve the pellets. The pellets were cut at 5 μm in thickness using a cryostat sectioning (Thermo Scientific, USA). The scaffold samples were fixed in 10% neutral formalin overnight. The samples were processed and embedded in paraffin wax. They were sectioned 5 μm. All samples were stained using the following techniques.

#### Alcian blue staining

To evaluate sericin’s proliferative and differentiative properties, alcian blue 8GX (Sigma-Aldrich, USA) was applied to assess glycosaminoglycans (GAGs) in the pellet samples. The pellet sections in all groups on days 7, 14, 21, and 28 were hydrated with 100, 95, and 70% ethanol for 2 min in each concentration. They were stained with 1% alcian blue 8GX (Sigma, USA) in 1% acetic acid (pH 2.5) for 30 min at room temperature. Afterward, they were dehydrated with a series of ethanol gradients and mounted with DEPEX (Electron Microscopy Sciences, USA). The pellet sections were examined under a microscope (BX51, Olympus®) and digital camera (DP70, Olympus®).

The positive cytological labeled area (blue area) was semi-quantified based on the H-score (a percentage of expression area; 0–100% × an intensity staining score; 0–3). The percentage of expression area was localized using ImageJ (NIH, USA), an image analysis program. The intensity staining score was graded as 0 = negative staining, 1 = low-intensity staining, 2 = moderate-intensity staining, and 3 = high-intensity staining.

#### Immunohistochemical staining

To investigate the proliferative, differentiative, and anti-inflammatory properties of sericin on chondrogenic cells, immunohistochemical staining was performed to immunolocalize cartilage-specific markers (COL2A1, ALP, COL X, aggrecan, and MMP-1) and anti-inflammatory markers (IL-1β and TNF-α). Sections of pellets from days 7, 14, 21, and 28 and scaffold samples were deparaffinized and hydrated. Heat-induced antigen retrieval in a citrate buffer (pH 6.0) was applied using a microwave for 10 min. The sections were immersed in 0.5% hydrogen peroxide in methanol for 5 min. The sections were incubated with 2% bovine serum albumin for 10 min to block nonspecific binding. Then, rabbit polyclonal antibodies (MyBioSource®, USA) were added to the section and incubated for 1 h. The sections were incubated with polymer HRP anti‐mouse/rabbit (DAKO, Denmark) labeling for 30 min and visualized using 3,3’-diaminobenzidine (DAB) (DAKO, Denmark) for 3 min. They were counterstained with hematoxylin, dehydrated with a series of ethanol, and mounted with DEPEX (Electron Microscopy Sciences, USA). The sections were examined under a microscope (BX51, Olympus®) and digital camera (DP70, Olympus®). The immunocytological labeled area was semiquantified based on the H-score as mentioned above.

### Proteomic analysis

The proteomic technique was performed in this study to demonstrate the effect of sericin on protein expressions and the specific mechanisms in chondrocyte proliferation and differentiation.

#### Pellets protein extraction

The 15 pellets in each group of 28-day positive control and CM50 were solubilized in 500 μl of lysis buffer containing 1% sodium dodecyl sulfate (SDS), 1% Triton-X, and 0.5% sodium chloride (NaCl), using ultrasonication on ice for 2 min. The samples were centrifuged at 10,000 × g for 10 min at 4 °C. The supernatant was measured the total protein concentration using a Bradford protein assay (Bio-Rad®, USA).

#### Label-free proteomic analysis

The proteins were centrifuged and precipitated in ice-cold acetone (1:5 *v*/*v*). After precipitation, the protein pellet was reconstituted in 0.25% RapidGest SF (Waters™, USA) in 15 mM ammonium bicarbonate (Sigma-Aldrich, USA.). 60 µg of protein in each group was subjected to gel-free digestion. Then, sulfhydryl bond reduction and sulfhydryl alkylation were performed using 5 mM DTT (Sigma-Aldrich, USA) in 15 mM ammonium bicarbonate at 72 °C for 1 h and adding IAA (Sigma-Aldrich, USA) in 15 mM ammonium bicarbonate at room temperature for 30 min in the dark, respectively. The solution was desalted by a Zeba Spin Desalting Column (Thermo Scientific, USA), digested with trypsin (Promega Co., Madison, WI, USA), and incubated at 37 °C for 3 h. The digested solution was dried and reconstituted in 0.1% formic acid before being subjected to LC–MS/MS. The experiment was conducted in 3-biological replications.

The LC–MS/MS spectrum data were collected in the positive mode with an HF-X Hybrid Quadrupole-Orbitrap™ Mass Spectrometer combined with an EASY-nLC1000 nano-LC system equipped with a nano-C18 column. Mobile phase A comprises 0.1% formic acid, and mobile phase B comprises 90% acetonitrile with 0.1% formic acid. The samples were loaded into an analytical C_18_ column.

#### Protein identification and quantification

The raw mass spectra (.raw file) were analyzed using MaxQuant v2.4.2.0, and proteins were identified using the UniProt protein database (www.uniprot.org, organism: *Mus musculus*). Next, protein identification and quantification were performed: MS tolerance, 20 ppm; MS/MS tolerance, 0.05 Da; digestion enzyme, trypsin; fixed modification, cysteine carbamidomethylation; and variable modification, methionine oxidation. The false discovery rate was 1% for peptides and protein identification. The proteins that had significant levels (*p* < 0.05) and a fold change ≥ 2 were categorized as biological functions using the UniProt database (www.uniprot.org, organism: *Mus musculus*) and PubMed. The associated proteins in chondrogenic proliferation and differentiation were investigated using the Reactome database biological pathways (www.reactome.org/PathwayBrowser, organism: *Mus musculus*).

### Immunogold labeling (TEM)

To examine the cytoskeletal ultrastructure associated with the proliferation and differentiation of chondrogenic cells, immunogold labeling was performed under transmission electron microscopy (TEM). The pellets 28-day neg control, Se50, pos control, and CM50 groups were fixed in 2.5% glutaraldehyde in 0.1 M SPB for 1 h and 1% osmium tetroxide in 0.1 M SPB for 1 h, respectively. In each concentration, the pellet samples were dehydrated in 30, 50, and 70% ethanol for 10 min. They were infiltrated with an LR white resin series (EMS®, USA). The samples were transferred into a mold embedding capsule, embedded in pure LR white resin (EMS®, USA), and polymerized at 60 °C for 48 h. The pellet samples were cut into a 100 nm and incubated with primary antibodies, including rabbit polyclonal anti-F-actin, -β-tubulin, and -COL2A1 (MyBioSource®, USA) for 1 h. After incubation, goat antirabbit conjugate gold particle 10 nm and goat antimouse conjugate gold particle 15 nm were added to the pellet section for 1 h. They enhanced the contrast of gold particles using a silver enhancement (Aurion R-Gent SE-EM kit, USA) for 30 min. The samples were stained with uranyl acetate and lead citrate before being investigated under the transmission electron microscope (HT7700; Hitachi, Japan). The images were captured at matrix area 10 fields/section, and each field was counted with the number of labeled gold particles.

### Quantitative Real‐Time polymerase chain reaction (RT‐qPCR)

Gene expression analysis was performed to identify the specific genes involved in the proliferation, differentiation, and inflammation of chondrocytes using RT-qPCR. All the samples were collected in triplicate. They were preserved in RNA*later* solution (Thermo Scientific, USA) to prevent degradation of RNA.

#### RNA extraction

The RNA samples were extracted using the RNeasy Mini Kit (Qiagen, Canada) following the company’s protocol. Briefly, the samples were ground homogeneously in 600 μl of lysis buffer. They were centrifuged and supernatant was transferred into a spin column tube. The RNA samples were bound in the column and washed several times with buffer. The RNA samples were then eluted into 35 μl rNase-free water. The RNA concentrations were measured by a NanoDrop™ 2000/2000c spectrophotometer (Thermo Scientific, USA).

#### RT‐qPCR

To explore the gene expression of chondrogenic proliferation and differentiation markers (*SOX-9, COL2A1, ALP, Aggecan, PCNA*), inflammatory markers (*IL-1β*, *TNF-α*, and *MMP-13*), and signaling pathways (*Smad1, Smad2, Smad3, BMP2*, and *BMP4*), RT-qPCR assay was performed using iTaq™ Universal SYBR Green Supermix (BIO‐RAD, USA). Primers were used in this study, shown in Supplementary Table S5, and all samples were processed in the CFX96 Touch™ Real-time PCR detector. Individual gene expression was calculated using the 2^−∆∆Ct^ method. The GAPDH was used as a reference gene for accurate data normalization.

### Statistical analysis

The statistical analysis used GraphPad Prism version 10.0 and SPSS version 23. The level of significant difference was presented as * = *p* < 0.05, ** = *p* < 0.01, *** = *p* < 0.001, and **** = *p* < 0.0001. All data were calculated by mean ± SEM with ANOVA and independent t-test.

### Supplementary Information

Supplementary Information 1.

Supplementary Information 2.

Supplementary Information 3.

Supplementary Information 4.

Supplementary Information 5.

## Data Availability

The data sets used in the current study may be shared upon a reasonable request to Sumate Ampawong, Ph.D.

## References

[1] Charlier E (2019). Chondrocyte dedifferentiation and osteoarthritis (OA). Biochem. Pharmacol..

[2] Emery CA (2019). Establishing outcome measures in early knee osteoarthritis. Nat. Rev. Rheumatol..

[3] Yao Q (2023). Osteoarthritis: Pathogenic signaling pathways and therapeutic targets. Signal Transduct. Target Ther..

[4] Disease GBD, Injury I, Prevalence C (2018). Global, regional, and national incidence, prevalence, and years lived with disability for 354 diseases and injuries for 195 countries and territories, 1990–2017: A systematic analysis for the Global Burden of Disease Study 2017. Lancet.

[5] Cui A (2020). Global, regional prevalence, incidence and risk factors of knee osteoarthritis in population-based studies. EClinicalMedicine.

[6] Bacenkova D, Trebunova M, Demeterova J, Zivcak J (2023). Human chondrocytes, metabolism of articular cartilage, and strategies for application to tissue engineering. Int. J. Mol. Sci..

[7] Akkiraju H, Nohe A (2015). Role of chondrocytes in cartilage formation, progression of osteoarthritis and cartilage regeneration. J. Dev. Biol..

[8] Li Y, Nie J, Deng C, Li H (2023). P-15 promotes chondrocyte proliferation in osteoarthritis by regulating SFPQ to target the Akt-RUNX2 axis. J. Orthop. Surg. Res..

[9] Ratneswaran A, Kapoor M (2021). Osteoarthritis year in review: Genetics, genomics, epigenetics. Osteoarthr. Cartil..

[10] Troeberg L, Nagase H (1824). Proteases involved in cartilage matrix degradation in osteoarthritis. Biochim. Biophys. Acta.

[11] Lo MY, Kim HT (2004). Chondrocyte apoptosis induced by collagen degradation: Inhibition by caspase inhibitors and IGF-1. J. Orthop. Res..

[12] Aramwit P, Palapinyo S, Srichana T, Chottanapund S, Muangman P (2013). Silk sericin ameliorates wound healing and its clinical efficacy in burn wounds. Arch. Dermatol. Res..

[13] Mondal M, Trivedy K, Kumar N (2006). The silk proteins, sericin and fibroin in silkworm, *Bombyx mori* Linn.,-a review. J. Entomol..

[14] Guo K (2022). Identification and characterization of sericin5 reveals non-cocoon silk sericin components with high beta-sheet content and adhesive strength. Acta Biomater..

[15] Aramwit P, Kanokpanont S, Nakpheng T, Srichana T (2010). The effect of sericin from various extraction methods on cell viability and collagen production. Int. J. Mol. Sci..

[16] Aramwit P, Sangcakul A (2007). The effects of sericin cream on wound healing in rats. Biosci. Biotechnol. Biochem..

[17] Aramwit P, Towiwat P, Srichana T (2013). Anti-inflammatory potential of silk sericin. Nat. Prod. Commun..

[18] Dinescu S (2013). Biocompatibility assessment of novel collagen-sericin scaffolds improved with hyaluronic Acid and chondroitin sulfate for cartilage regeneration. Biomed. Res. Int..

[19] Qi C (2018). Photo-crosslinkable, injectable sericin hydrogel as 3D biomimetic extracellular matrix for minimally invasive repairing cartilage. Biomaterials.

[20] Song J, Baek IJ, Chun CH, Jin EJ (2018). Dysregulation of the NUDT7-PGAM1 axis is responsible for chondrocyte death during osteoarthritis pathogenesis. Nat. Commun..

[21] Adachi T (2022). Three-dimensional culture of cartilage tissue on nanogel-cross-linked porous freeze-dried gel scaffold for regenerative cartilage therapy: A vibrational spectroscopy evaluation. Int. J. Mol. Sci..

[22] Alcaide-Ruggiero L, Molina-Hernandez V, Granados MM, Dominguez JM (2021). Main and minor types of collagens in the articular cartilage: The role of collagens in repair tissue evaluation in chondral defects. Int. J. Mol. Sci..

[23] Thomas JD (2014). Rab1A is an mTORC1 activator and a colorectal oncogene. Cancer Cell.

[24] Chen J, Long F (2018). mTOR signaling in skeletal development and disease. Bone Res..

[25] Liu S (2021). Periostin regulates osteogenesis of mesenchymal stem cells from ovariectomized rats through actions on the ILK/Akt/GSK-3beta Axis. Genet. Mol. Biol..

[26] Chen KS (2010). Periostin expression distinguishes between light and dark hypertrophic chondrocytes. Int. J. Biochem. Cell Biol..

[27] Bottcher RT (2009). Profilin 1 is required for abscission during late cytokinesis of chondrocytes. EMBO J..

[28] Marsich E (2008). Galectin-1 in cartilage: Expression, influence on chondrocyte growth and interaction with ECM components. Matrix Biol..

[29] Silva JC (2020). Glycosaminoglycan remodeling during chondrogenic differentiation of human bone marrow-/synovial-derived mesenchymal stem/stromal cells under normoxia and hypoxia. Glycoconj. J..

[30] Venkatesan N (2012). Xylosyltransferase-I regulates glycosaminoglycan synthesis during the pathogenic process of human osteoarthritis. PLoS One.

[31] Cao Z, Dou C, Dong S (2017). Curcumin inhibits chondrocyte hypertrophy of mesenchymal stem cells through IHH and notch signaling pathways. Chem. Pharm. Bull..

[32] Fu R (2019). The Effects of leptin on the proliferation and differentiation of primary chondrocytes in vitro and cartilage regeneration in vivo. ACS Biomater. Sci. Eng..

[33] Caron MM (2013). Hypertrophic differentiation during chondrogenic differentiation of progenitor cells is stimulated by BMP-2 but suppressed by BMP-7. Osteoarthr. Cartil..

[34] Kudo T (2017). Supplemented chondroitin sulfate and hyaluronic acid suppress mineralization of the chondrogenic cell line, ATDC5, via direct inhibition of alkaline phosphatase. Biol. Pharm. Bull..

[35] Goldring MB (2012). Chondrogenesis, chondrocyte differentiation, and articular cartilage metabolism in health and osteoarthritis. Ther. Adv. Musculoskelet. Dis..

[36] Ni Y (2019). Chondrocytes cultured in silk-based biomaterials maintain function and cell morphology. Int. J. Artif. Organs.

[37] Chomchalao P, Pongcharoen S, Sutheerawattananonda M, Tiyaboonchai W (2013). Fibroin and fibroin blended three-dimensional scaffolds for rat chondrocyte culture. Biomed. Eng. Online.

[38] Teimourinejad A (2020). Chondrogenic activity of two herbal products; pomegranate fruit extract and avocado/soybean unsaponifiable. Res. Pharm. Sci..

[39] Katani M, Zolfaghari B, Soleimani M, Valiani A, Hashemibeni B (2017). The effect of pomegranate extract on producing type II collagen in differentiation of adipose-derived stem cells into chondrocytes. J. Isfahan Med. Sch..

[40] Caron MMJ (2020). Aggrecan and COMP improve periosteal chondrogenesis by delaying chondrocyte hypertrophic maturation. Front. Bioeng. Biotechnol..

[41] Blain EJ (2009). Involvement of the cytoskeletal elements in articular cartilage homeostasis and pathology. Int. J. Exp. Pathol..

[42] Binarova P, Tuszynski J (2019). Tubulin: Structure, functions and roles in disease. Cells.

[43] Capin-Gutierrez N, Talamas-Rohana P, Gonzalez-Robles A, Lavalle-Montalvo C, Kouri JB (2004). Cytoskeleton disruption in chondrocytes from a rat osteoarthrosic (OA) -induced model: Its potential role in OA pathogenesis. Histol. Histopathol..

[44] Leung VY (2011). SOX9 governs differentiation stage-specific gene expression in growth plate chondrocytes via direct concomitant transactivation and repression. PLoS Genet.

[45] Oh CD (2014). SOX9 regulates multiple genes in chondrocytes, including genes encoding ECM proteins, ECM modification enzymes, receptors, and transporters. PLoS One.

[46] Voga M, Drnovsek N, Novak S, Majdic G (2019). Silk fibroin induces chondrogenic differentiation of canine adipose-derived multipotent mesenchymal stromal cells/mesenchymal stem cells. J. Tissue Eng..

[47] Aigner T, Stove J (2003). Collagens–major component of the physiological cartilage matrix, major target of cartilage degeneration, major tool in cartilage repair. Adv. Drug Deliv. Rev..

[48] Yassin AM (2020). COL2A1 and Caspase-3 as promising biomarkers for osteoarthritis prognosis in an *Equus asinus* model. Biomolecules.

[49] Henrotin YE (2006). Avocado/soybean unsaponifiables prevent the inhibitory effect of osteoarthritic subchondral osteoblasts on aggrecan and type II collagen synthesis by chondrocytes. J. Rheumatol..

[50] Schonenberger F, Deutzmann A, Ferrando-May E, Merhof D (2015). Discrimination of cell cycle phases in PCNA-immunolabeled cells. BMC Bioinform..

[51] Kobayashi, T., Young, C., Zhou, W. & Rhee, E. P. Reduced glycolysis links resting zone chondrocyte proliferation in the growth plate. *bioRxiv*. 10.1101/2023.01.18.524550 (2023).

[52] Kalucka J (2015). Metabolic control of the cell cycle. Cell Cycle.

[53] Jiang X, Sun Q, Li H, Li K, Ren X (2014). The role of phosphoglycerate mutase 1 in tumor aerobic glycolysis and its potential therapeutic implications. Int. J. Cancer.

[54] Li N, Liu X (2020). Phosphoglycerate mutase 1: Its glycolytic and non-glycolytic roles in tumor malignant behaviors and potential therapeutic significance. Onco Targets Ther..

[55] Xu Z (2016). The diagnostic value and functional roles of phosphoglycerate mutase 1 in glioma. Oncol. Rep..

[56] Tay L (2015). Proteomics of chondrogenesis: A review. JUMMEC.

[57] Ruiz-Romero C (2008). Proteomic analysis of human osteoarthritic chondrocytes reveals protein changes in stress and glycolysis. Proteomics.

[58] Shen J, Li S, Chen D (2014). TGF-beta signaling and the development of osteoarthritis. Bone Res..

[59] Van der Kraan PM, Davidson EB, Blom A, Van den Berg WB (2009). TGF-beta signaling in chondrocyte terminal differentiation and osteoarthritis: Modulation and integration of signaling pathways through receptor-Smads. Osteoarthr. Cartil..

[60] Chavez RD, Coricor G, Perez J, Seo HS, Serra R (2017). SOX9 protein is stabilized by TGF-β and regulates PAPSS2 mRNA expression in chondrocytes. Osteoarthr. Cartil..

[61] Xiao L (2018). TGF-beta/SMAD signaling inhibits intermittent cyclic mechanical tension-induced degeneration of endplate chondrocytes by regulating the miR-455-5p/RUNX2 axis. J. Cell Biochem..

[62] Sun M, Lu Z, Cai P, Zheng L, Zhao J (2020). Salidroside enhances proliferation and maintains phenotype of articular chondrocytes for autologous chondrocyte implantation (ACI) via TGF-β/Smad3 Signal. Biomed. Pharmacother..

[63] Wang W (2016). Smad2 and Smad3 regulate chondrocyte proliferation and differentiation in the growth plate. PLOS Genet..

[64] Chen H (2021). Molecular mechanisms of chondrocyte proliferation and differentiation. Front. Cell Dev. Biol..

[65] Li TF (2006). Smad3-deficient chondrocytes have enhanced BMP signaling and accelerated differentiation. J. Bone Miner. Res..

[66] Zhang H, Cai D, Bai X (2020). Macrophages regulate the progression of osteoarthritis. Osteoarthr. Cartil..

[67] Aramwit P (2023). Sericin coated thin polymeric films reduce keratinocyte proliferation via the mTOR pathway and epidermal inflammation through IL17 signaling in psoriasis rat model. Sci. Rep..

[68] Sun Y (2023). Multi-omics integration to reveal the mechanism of sericin inhibiting LPS-induced inflammation. Int. J. Mol. Sci..

[69] Mathy-Hartert M (2009). Curcumin inhibits pro-inflammatory mediators and metalloproteinase-3 production by chondrocytes. Inflamm. Res..

[70] Au RY, Al-Talib TK, Au AY, Phan PV, Frondoza CG (2007). Avocado soybean unsaponifiables (ASU) suppress TNF-alpha, IL-1beta, COX-2, iNOS gene expression, and prostaglandin E2 and nitric oxide production in articular chondrocytes and monocyte/macrophages. Osteoarthr. Carti..

[71] Xu X (2017). Danshen attenuates osteoarthritis-related cartilage degeneration through inhibition of NF-kappaB signaling pathway in vivo and in vitro. Biochem. Cell Biol..

[72] Zhang J (2022). Icariin: A potential molecule for treatment of knee osteoarthritis. Front. Pharmacol..

[73] Ashraaf S (2023). Synergistic effect of silk sericin and curcumin to treat an inflammatory condition. J. Burn Care Res..

[74] Richardson DW, Dodge GR (2003). Dose-dependent effects of corticosteroids on the expression of matrix-related genes in normal and cytokine-treated articular chondrocytes. Inflamm. Res..

[75] Huebner KD, Shrive NG, Frank CB (2014). Dexamethasone inhibits inflammation and cartilage damage in a new model of post-traumatic osteoarthritis. J. Orthop. Res..

[76] Cheleschi S (2021). A combination of celecoxib and glucosamine sulfate has anti-inflammatory and chondroprotective effects: Results from an in vitro study on human osteoarthritic chondrocytes. Int. J. Mol. Sci..

[77] Veronese N (2020). Glucosamine sulphate: An umbrella review of health outcomes. Ther. Adv. Musculoskelet. Dis..

[78] Derfoul A, Miyoshi AD, Freeman DE, Tuan RS (2007). Glucosamine promotes chondrogenic phenotype in both chondrocytes and mesenchymal stem cells and inhibits MMP-13 expression and matrix degradation. Osteoarthr. Cartil..

[79] Ampawong S, Aramwit P (2017). In vivo safety and efficacy of sericin/poly(vinyl alcohol)/glycerin scaffolds fabricated by freeze-drying and salt-leaching techniques for wound dressing applications. J. Bioact. Compat. Polym..

[80] Lopez-Garcia J, Lehocky M, Humpolicek P, Saha P (2014). HaCaT keratinocytes response on antimicrobial atelocollagen substrates: Extent of cytotoxicity, cell viability and proliferation. J. Funct. Biomater..

[81] Tare RS, Howard D, Pound JC, Roach HI, Oreffo RO (2005). Tissue engineering strategies for cartilage generation–micromass and three dimensional cultures using human chondrocytes and a continuous cell line. Biochem. Biophys. Res. Commun..

