# Supplementary material for: Sericin promotes chondrogenic proliferation and differentiation via glycolysis and Smad2/3 TGF-β signaling inductions and alleviates inflammation in three-dimensional models
Source: Sci Rep. 2024 May 21;14:11553. doi: 10.1038/s41598-024-62516-y (PMC11109159; doi:10.1038/s41598-024-62516-y)
Supplement: Supplementary file 1 — Supplementary Information 1. [file 41598_2024_62516_MOESM1_ESM.pdf]

Table S1. The statistical analysis of Pearson correlations in pellet groups with COL2A1, ALP, COL X, and aggrecan markers from day 7 to day 28.

Correlations

		Collagen2a1	ALP	Collagen X	Aggrecan
Collagen2a1	Pearson Correlation	1	.062	.592**	.231*
	Sig. (2-tailed)		.563	.000	.019
	N	102	90	91	102
ALP	Pearson Correlation	.062	1	.377**	-.159
	Sig. (2-tailed)	.563		.000	.132
	N	90	91	87	91
Collagen10a1	Pearson Correlation	.592**	.377**	1	-.049
	Sig. (2-tailed)	.000	.000		.637
	N	91	87	94	94
Aggrecan	Pearson Correlation	.231*	-.159	-.049	1
	Sig. (2-tailed)	.019	.132	.637	
	N	102	91	94	184

**. Correlation is significant at the 0.01 level (2-tailed).

*. Correlation is significant at the 0.05 level (2-tailed).
