# Supplementary material for: Sericin promotes chondrogenic proliferation and differentiation via glycolysis and Smad2/3 TGF-β signaling inductions and alleviates inflammation in three-dimensional models
Source: Sci Rep. 2024 May 21;14:11553. doi: 10.1038/s41598-024-62516-y (PMC11109159; doi:10.1038/s41598-024-62516-y)
Supplement: Supplementary file 2 — Supplementary Information 2. [file 41598_2024_62516_MOESM2_ESM.pdf]

Table S2. The 41 downregulation proteins which no statistically significant difference.

Protein accession	p-value	Protein name	Fold change
H2A1E_MOUSE	0.20909208	Histone H2A type 1-E	0.139
ARP3_MOUSE	0.159042711	Actin-related protein 3	0.670
PFKAP_MOUSE	0.194337626	Phosphofructokinase	0.691
IL5RA_MOUSE	0.07945278	Interleukin-5 receptor subunit alpha	0.753
TCPA_MOUSE	0.59934328	T-complex protein 1 subunit alpha	0.771
H14_MOUSE	0.83463356	Histone H1.4	0.777
SMD2_MOUSE	0.317952369	Small nuclear ribonucleoprotein Sm D2	0.782
TTC16_MOUSE	0.534070926	Tetratricopeptide repeat protein 16	0.785
NUP85_MOUSE	0.601703832	Nuclear pore complex protein Nup85	0.810
DAD1_MOUSE	0.511658624	Dolichyl-diphosphooligosaccharide--protein glycosyltransferase subunit DAD1	0.843
BAF_MOUSE	0.071619853	Barrier-to-autointegration factor	0.849
RACK1_MOUSE	0.615531727	Receptor of activated protein C kinase 1	0.856
RL24_MOUSE	0.730081952	60S ribosomal protein L24	0.872
RCN2_MOUSE	0.662732279	Reticulocalbin-2	0.883
HMGB1_MOUSE	0.793670412	High mobility group protein 1	0.885
SYRC_MOUSE	0.682023872	Arginine--tRNA ligase, cytoplasmic	0.886
RAC1_MOUSE	0.655853512	Ras-related C3 botulinum toxin substrate 1	0.894
STMN1_MOUSE	0.714014199	Stathmin	0.898
VDAC3_MOUSE	0.33875163	Voltage-dependent anion channel 3	0.904
RD23A_MOUSE	0.691539653	UV excision repair protein RAD23 homolog A	0.905
THOC4_MOUSE	0.836914853	THO complex subunit 4	0.917
MFGM_MOUSE	0.790094916	Lactadherin	0.924
HINT1_MOUSE	0.787407589	Adenosine 5-monophosphoramidase HINT1	0.928
ROA3_MOUSE	0.775736603	Ribonucleoprotein heterogeneous nuclear ribonucleoprotein A3	0.931
IF172_MOUSE	0.751797578	Intraflagellar transport protein 172 homolog	0.933
SSRD_MOUSE	0.917396075	Translocon-associated protein subunit delta isoform 4	0.937
6PGD_MOUSE	0.774843056	6-phosphogluconate dehydrogenase, decarboxylating	0.944
RL13_MOUSE	0.881990313	60S ribosomal protein L13	0.948
VAPA_MOUSE	0.805976379	Vesicle-associated membrane protein-associated protein A	0.954
SSRP1_MOUSE	0.879861989	FACT complex subunit SSRP1	0.958
FINC_MOUSE	0.936008027	Fn receptor beta	0.970
THIL_MOUSE	0.828635414	Acetyl-CoA acetyltransferase, mitochondrial	0.972
RL34_MOUSE	0.938061121	60S ribosomal protein L34	0.975
DDX17_MOUSE	0.884483427	Probable ATP-dependent RNA helicase DDX17	0.976
F193A_MOUSE	0.855587105	Protein FAM193A	0.977
AP1M1_MOUSE	0.950700204	AP-1 complex subunit mu-1	0.980
ACTN4_MOUSE	0.944663048	Alpha-actinin-4	0.981
PI51C_MOUSE	0.909309823	Phosphatidylinositol 4-phosphate 5-kinase type-1 gamma	0.983
HAP28_MOUSE	0.983192917	28 kDa heat- and acid-stable phosphoprotein	0.990

E5D6T0_MOUSE	0.990863625	Bromodomain PHD finger transcription factor splice variant	0.998
OSTA_MOUSE	0.997606943	Organic solute transporter subunit alpha	0.999
