# Supplementary material for: Sericin promotes chondrogenic proliferation and differentiation via glycolysis and Smad2/3 TGF-β signaling inductions and alleviates inflammation in three-dimensional models
Source: Sci Rep. 2024 May 21;14:11553. doi: 10.1038/s41598-024-62516-y (PMC11109159; doi:10.1038/s41598-024-62516-y)
Supplement: Supplementary file 3 — Supplementary Information 3. [file 41598_2024_62516_MOESM3_ESM.pdf]

Table S3. The 106 significant proteins with classified into 5 groups; 1. proliferation 22 proteins, 2. differentiation 18 proteins, 3. extracellular matrix 16 proteins, 4. cytoskeleton 9 proteins, and other functions 53 proteins.

p-value	Biological function groups	Protein name	Fold change
5.03415147E-05	1	Phosphoglycerate mutase 1	18.097
1.93850871E-05	1	Triosephosphate isomerase	14.460
2.00387615E-03	1	Transitional endoplasmic reticulum ATPase	6.729
3.09761732E-02	1	Lactate dehydrogenase-A	6.181
1.56364200E-04	1	Nucleophosmin 1	5.886
1.71369374E-03	1	Tyrosine 3-monooxygenase/tryptophan 5-monooxygenase activation protein, zeta polypeptide	5.664
8.27849186E-05	1	Annexin A1	5.586
3.28502362E-04	1	Phosphoglycerate kinase 1	5.109
1.34168416E-03	1	Alpha-enolase	4.718
1.05029192E-02	1	Glyceraldehyde-3-phosphate dehydrogenase	4.178
6.14293806E-04	1	Pyruvate kinase PKM	3.868
2.28387409E-02	1	Fructose-bisphosphate aldolase A	3.477
3.14866879E-03	1	Ribosomal protein S3	3.466
2.38944056E-03	1	Stress-70 protein, mitochondrial	3.379
6.78385943E-03	1	Lamina-associated polypeptide 2 isoform alpha	2.142
4.43972243E-04	1	Acidic leucine-rich nuclear phosphoprotein 32 family member B	2.062
3.70369461E-05	2	Galectin-1	10.770
6.38896279E-04	2	Annexin A5	7.917
8.75915926E-04	2	Calreticulin	7.840
1.99965323E-04	2	Annexin A8	7.327
3.12258938E-05	2	Moesin	6.034
3.06979032E-03	2	Glycosylation-inhibiting factor	5.474
7.53789445E-03	2	Coagulation factor XIII A chain	4.913
9.28271819E-05	2	Peptidyl-prolyl cis-trans isomerase A or cyclophilin A	4.759
7.52810689E-05	2	Cell division control protein 42 homolog	4.449
7.70061385E-03	2	Heat shock protein HSP 90-beta	4.117
1.08200581E-04	2	Glutathione S-transferase P1	3.619
1.06257389E-02	3	Procollagen, type XII, alpha 1	12.989
7.61693720E-03	3	Collagen alpha-3(VI) chain	10.729
5.49180409E-04	3	Collagen alpha-2(VI) chain	7.505
2.31687905E-02	3	Serpin H1	6.585
3.74897928E-04	3	Collagen alpha-1(XI) chain	5.392
1.43200972E-02	3	Collagen alpha-1(VI) chain	5.204
2.12447851E-03	3	Prolyl 4-hydroxylase subunit alpha-1	4.486
2.23297630E-04	3	Fibromodulin	3.920
1.82801530E-05	3	Biglycan	3.565
1.24327964E-02	3	Sushi repeat-containing protein SRPX2	3.332
1.66551969E-03	3	Aggrecan core protein or Cartilage-specific proteoglycan core protein	2.779
1.01406213E-03	3	Procollagen-proline, 2-oxoglutarate 4-dioxygenase (proline 4-hydroxylase), alpha II	2.379
2.57189044E-04	4	Tubulin beta-5 chain	5.817

1.90974036E-04	4	Tubulin, alpha 1B	4.763
1.42405373E-02	4	Cytoskeleton-associated protein 4	4.523
3.58537014E-03	4	Myosin-9	4.402
2.28086220E-02	4	Macrophage-capping protein	3.867
4.19872918E-03	4	Actin, cytoplasmic 1 (Actin, beta)	3.856
2.87858099E-04	4	Filamin-A	3.182
1.35224589E-03	5	Polyubiquitin C	16.848
4.23948939E-03	5	Peroxiredoxin-5, mitochondrial isoform 1 precursor	9.373
1.58582241E-04	5	Histone H2AX	9.076
1.47560672E-04	5	Eukaryotic translation elongation factor 2	8.922
6.34728875E-04	5	Heterogeneous nuclear ribonucleoproteins A2/B1 isoform 3	8.739
2.92396702E-02	5	Rho GDP dissociation inhibitor (GDI) alpha	8.134
2.31240746E-02	5	Prolyl 4-hydroxylase, beta polypeptide	7.830
1.86868889E-03	5	ATP synthase subunit beta, mitochondrial	7.256
1.94979811E-02	5	Heat shock protein 5	6.071
4.27208016E-04	5	40S ribosomal protein S7	5.755
6.15665848E-03	5	Voltage-dependent anion-selective channel protein 2	5.716
1.33825865E-03	5	40S ribosomal protein S16	5.401
1.07510839E-03	5	60S acidic ribosomal protein P0	5.385
3.37279085E-02	5	ATP synthase alpha subunit	5.146
4.51036659E-05	5	Malate dehydrogenase, mitochondrial	5.141
9.53763759E-04	5	Succinyl-CoA:3-ketoacid coenzyme A transferase 1, mitochondrial	4.992
2.10985399E-03	5	3-hydroxyacyl-CoA dehydrogenase type-2	4.992
3.42622096E-03	5	Peptidyl-prolyl cis-trans isomerase B	4.839
2.47909151E-02	5	Protein disulfide-isomerase A3	4.732
1.35199343E-02	5	ADP-ribosylation factor 4	4.650
6.88267844E-07	5	Heterogeneous nuclear ribonucleoprotein Q	4.627
1.04776673E-03	5	Reticulocalbin-3	4.402
2.03886857E-02	5	Nucleoside diphosphate kinase B	4.304
1.25732887E-02	5	60S ribosomal protein L4	4.087
1.42841674E-02	5	Elongation factor 1-beta	4.020
3.00700213E-03	5	60S ribosomal protein L9	3.866
1.38998030E-03	5	Dolichyl-diphosphooligosaccharide--protein glycosyltransferase subunit 1	3.855
1.80269094E-04	5	Nucleolin	3.580
5.54816756E-03	5	Prosaposin	3.526
7.53670535E-03	5	40S ribosomal protein S8	3.406
1.03894745E-02	5	Dolichyl-diphosphooligosaccharide--protein glycosyltransferase 48 kDa subunit	3.364
2.77036564E-03	5	Polypyrimidine tract binding protein 1	3.325
1.82552405E-02	5	Leucine-rich repeat-containing protein 59	3.261
2.59134053E-04	5	Heterogeneous nuclear ribonucleoprotein M	3.240
1.17988947E-02	5	Alanine--tRNA ligase, cytoplasmic	3.222
1.82183954E-02	5	Elongation factor 1-gamma	3.081
2.15355600E-02	5	60S ribosomal protein L6	2.917
1.44529400E-02	5	Ferritin light chain	2.839
2.07880385E-02	5	Peptidyl-prolyl cis-trans isomerase FKBP10	2.832

2.02168924E-02	5	Peroxiredoxin-2	2.673
1.29701292E-02	5	Heterogeneous nuclear ribonucleoprotein H	2.562
1.65519133E-02	5	Clathrin, heavy polypeptide (Hc)	2.509
4.39625948E-03	5	Ubiquitin-conjugating enzyme E2 L3	2.493
1.88839304E-02	5	60S ribosomal protein L22	2.240
1.41661430E-02	5	Heterogeneous nuclear ribonucleoprotein L	2.166
1.91975818E-02	5	14-3-3 protein beta/alpha	2.153
5.39559339E-03	5	Acidic leucine-rich nuclear phosphoprotein 32 family member A	2.129
3.79772538E-03	5	40S ribosomal protein S18	2.117
1.85467630E-02	5	Syntenin-1	2.072
4.25039157E-03	5	Endoplasmic reticulum oxidoreductase alpha	2.002
6.14221911E-04	5	40S ribosomal protein SA	6.604
8.38197766E-04	1 and 2	Rab1A	11.682
1.74740376E-02	1 and 2	Phosphatidylethanolamine-binding protein 1	3.019
2.41755037E-04	1 and 4	Profilin 1	11.080
3.48996493E-03	1, 2, and 3	Thrombospondin 1	8.916
2.58989636E-03	1, 2, and 3	Fibronectin	3.666
1.38605688E-02	1, 2, and 5	Heat shock cognate 71 kDa protein	9.984
8.26494598E-05	2 and 3	Collagen-alpha-1 type X	12.169
2.12358223E-06	2 and 3	Periostin	11.503
1.69473202E-02	4 and 5	Heat shock protein beta-1	2.094
