# Supplementary material for: Sericin promotes chondrogenic proliferation and differentiation via glycolysis and Smad2/3 TGF-β signaling inductions and alleviates inflammation in three-dimensional models
Source: Sci Rep. 2024 May 21;14:11553. doi: 10.1038/s41598-024-62516-y (PMC11109159; doi:10.1038/s41598-024-62516-y)
Supplement: Supplementary file 4 — Supplementary Information 4. [file 41598_2024_62516_MOESM4_ESM.pdf]

Table S4. The statistical analysis of Pearson correlations in early inflammation with IL-1β, TNF-α, MMP-1, and COL2A1 markers at day 21.

Correlations

		IL-1β	TNF-α	MMP1	Collagen2a1
IL-1β	Pearson Correlation	1	.493**	.609**	-.177**
	Sig. (2-tailed)		.000	.000	.009
	N	221	203	208	214
TNF-α	Pearson Correlation	.493**	1	.535**	-.173*
	Sig. (2-tailed)	.000		.000	.016
	N	203	203	202	196
MMP1	Pearson Correlation	.609**	.535**	1	-.263**
	Sig. (2-tailed)	.000	.000		.000
	N	208	202	209	203
Collagen2a1	Pearson Correlation	-.177**	-.173*	-.263**	1
	Sig. (2-tailed)	.009	.016	.000	
	N	214	196	203	218

**. Correlation is significant at the 0.01 level (2-tailed).

*. Correlation is significant at the 0.05 level (2-tailed).
