# Supplementary material for: Sericin promotes chondrogenic proliferation and differentiation via glycolysis and Smad2/3 TGF-β signaling inductions and alleviates inflammation in three-dimensional models
Source: Sci Rep. 2024 May 21;14:11553. doi: 10.1038/s41598-024-62516-y (PMC11109159; doi:10.1038/s41598-024-62516-y)
Supplement: Supplementary file 5 — Supplementary Information 5. [file 41598_2024_62516_MOESM5_ESM.pdf]

Table S5. The mouse primers for RT-qPCR

Gene name	Primer sequences (5'-3')	Annealing temperature (°C)	Length (bp)	Reference
mGAPDH	FW: TCAACAGCAACTCCCACTCTTCCA<br>RV: ACCCTGTTGCTGTAGCCGTATTCA	60	115	[1]
mSOX-9	FW: AGGAAGCTGGCAGACCAGTA<br>RV: TCCACGAAGGGTCTCTTCTC	60	107	[2]
mCOL2A1	FW: GAGCAGCAAGAGCAAGGAAA<br>RV: CGGAGGAAAGTCATCTGGAC	60	126	[3]
mALP	FW: AATGAGGTCACATCCATCCTG<br>RV: CACCCGAGTGGTAGTCACAA	60	75	[4]
mAggrecan	FW: GGGACAGGGTGAGAAGTAAGG<br>RV: CTGGAGGCGAAGTAACCAAC	60	109	[3]
mPCNA	FW: TGCTCTGAGGTACCTGAACT<br>RV: TGCTTCCTCATCTTCAATCT	60	160	[5]
mRUNX2	FW: GTTCAACGATCTGAGATTTGTG<br>RV: GGATTTGTGAAGACTGTTATGG	60	78	[6]
mSmad1	FW: AAAGACCTGTGGCTTCCGTCT<br>RV: TTATCGTGGCTCCTTCGTCAG	60	103	[7]
mSmad2	FW: ATGTCGTCCATCTTGCCATTC<br>RV: AACCGTCCTGTTTTCTTTAGCTT	60	173	[8]
mSmad3	FW: CACAGCCACCATGAATTACG<br>RV: TGGAGGTAGAACTGGCGTCT	60	120	[9]
mBMP2	FW: ACACAGCTGGTCACAGATAA<br>RV: CTTCCGCTGTTTGTGTTTGG	60	109	[10]
mBMP4	FW: AACGTAGTCCCAAGCATCAC<br>RV: CGTCACTGAAGTCCACGTATAG	60	87	[10]
mIL-1β	FW: AAGATGAAGGGCTGCTTCCAAACC<br>RV: ATACTGCCTGCCTGAAGCTCTTGT	60	106	[11]
mTNF-α	FW: CGTCAGCCGATTTGCTATCT<br>RV: CGGACTCCGCAAAGTCTAAG	60	206	[11]
mMMP-13	FW: TGTTTGCAGAGCACTACTTGAA<br>RV: CAGTCACCTCTAAGCCAAAGAAA	60	132	[3]

References

1 Kobpornchai, P. *et al.* A novel cystatin derived from Trichinella spiralis suppresses macrophage-mediated inflammatory responses. *PLOS Neglected Tropical Diseases* **14**, e0008192, doi:10.1371/journal.pntd.0008192 (2020).

2 Wang, P. *et al.* Flavonoid Compound Icariin Activates Hypoxia Inducible Factor-1α in Chondrocytes and Promotes Articular Cartilage Repair. *PloS one* **11**, e0148372, doi:10.1371/journal.pone.0148372 (2016).

3 Wang, G. *et al.* TGFbeta attenuates cartilage extracellular matrix degradation via enhancing FBXO6-mediated MMP14 ubiquitination. *Ann Rheum Dis* **79**, 1111-1120, doi:10.1136/annrheumdis-2019-216911 (2020).

4 Liu, B. *et al.* A protocol for isolation and identification and comparative characterization of primary osteoblasts from mouse and rat calvaria. *Cell Tissue Bank* **20**, 173-182, doi:10.1007/s10561-019-09751-0 (2019).

5 Yan, C. *et al.* MicroRNA regulation associated chondrogenesis of mouse MSCs grown on polyhydroxyalkanoates. *Biomaterials* **32**, 6435-6444, doi:10.1016/j.biomaterials.2011.05.031 (2011).

6 Tsukamoto, I. *et al.* Activating types 1 and 2 angiotensin II receptors modulate the hypertrophic differentiation of chondrocytes. *FEBS Open Bio* **3**, 279-284, doi:10.1016/j.fob.2013.07.001 (2013).

7 He, Y. *et al.* Changes in osteogenic gene expression in hypertrophic chondrocytes induced by SIN-1. *Exp Ther Med* **16**, 609-618, doi:10.3892/etm.2018.6261 (2018).

8 He, Y. *et al.* Gene Expression Profile of Hypertrophic Chondrocytes Treated with H(2)O(2): A Preliminary Investigation. *Chin Med Sci J* **33**, 45-52, doi:10.24920/31801 (2018).

9 Chen, C. G., Thuillier, D., Chin, E. N. & Alliston, T. Chondrocyte-intrinsic Smad3 represses Runx2-inducible matrix metalloproteinase 13 expression to maintain articular cartilage and prevent osteoarthritis. *Arthritis Rheum* **64**, 3278-3289, doi:10.1002/art.34566 (2012).

10 Tabe, S. *et al.* Lysophosphatidylcholine acyltransferase 4 is involved in chondrogenic differentiation of ATDC5 cells. *Scientific Reports* **7**, 16701, doi:10.1038/s41598-017-16902-4 (2017).

11 Sun, T. *et al.* MiR-146a Aggravates LPS-Induced Inflammatory Injury by Targeting CXCR4 in the Articular Chondrocytes. *Cell Physiol Biochem* **44**, 1282-1294, doi:10.1159/000485488 (2017).
